# Chronic spinal cord injury repair by NT3-chitosan only occurs after clearance of the lesion scar

**DOI:** 10.1038/s41392-022-01010-1

**Published:** 2022-06-17

**Authors:** Can Zhao, Jia-Sheng Rao, Hongmei Duan, Peng Hao, Junkui Shang, Yubo Fan, Wen Zhao, Yudan Gao, Zhaoyang Yang, Yi Eve Sun, Xiaoguang Li

**Affiliations:** 1 Beijing Key Laboratory for Biomaterials and Neural Regeneration, Beijing Advanced Innovation Center for Biomedical Engineering, School of Biological Science and Medical Engineering, Beihang University, Beijing, 100083 China; 2 Institute of Rehabilitation Engineering, China Rehabilitation Science Institute, Beijing, 100068 China; 3 Department of Neurobiology, School of Basic Medical Sciences, Capital Medical University, Beijing, 100069 China; 4 Beijing Advanced Innovation Centre for Biomedical Engineering, Key Laboratory for Biomechanics and Mechanobiology of Chinese Education Ministry, School of Biological Science and Medical Engineering, Beihang University, Beijing, 10083 China; 5 School of Engineering Medicine, Beihang University, Beijing, 10083 China; 6 Shanghai Institute of Stem Cell Research and Clinical Translation, Shanghai East Hospital, Tongji University School of Medicine, Shanghai, 200120 China; 7 Department of Psychiatry and Biobehavioral Sciences, UCLA Medical School, Los Angeles, CA 90095 USA

**Keywords:** Regeneration and repair in the nervous system, Neurology

## Abstract

Spinal cord injury (SCI) is a severe damage usually leading to limb dysesthesia, motor dysfunction, and other physiological disability. We have previously shown that NT3-chitosan could trigger an acute SCI repairment in rats and non-human primates. Due to the negative effect of inhibitory molecules in glial scar on axonal regeneration, however, the role of NT3-chitosan in the treatment of chronic SCI remains unclear. Compared with the fresh wound of acute SCI, how to handle the lesion core and glial scars is a major issue related to chronic-SCI repair. Here we report, in a chronic complete SCI rat model, establishment of magnetic resonance-diffusion tensor imaging (MR-DTI) methods to monitor spatial and temporal changes of the lesion area, which matched well with anatomical analyses. Clearance of the lesion core via suction of cystic tissues and trimming of solid scar tissues before introducing NT3-chitosan using either a rigid tubular scaffold or a soft gel form led to robust neural regeneration, which interconnected the severed ascending and descending axons and accompanied with electrophysiological and motor functional recovery. In contrast, cystic tissue extraction without scar trimming followed by NT3-chitosan injection, resulted in little, if any regeneration. Taken together, after lesion core clearance, NT3-chitosan can be used to enable chronic-SCI repair and MR-DTI-based mapping of lesion area and monitoring of ongoing regeneration can potentially be implemented in clinical studies for subacute/chronic-SCI repair.

## Introduction

Spinal cord injury (SCI) is a debilitating medical condition often leading to paralysis, currently with no effective treatment. Failure of nerve regeneration in the central nervous system (CNS) root from attenuated axonal growth potential, which is intrinsic to CNS neurons, as well as inhibitory CNS injury environment, which is filled with myelin debris, inflammatory cytokines, and cells producing inhibitory molecules,^1–4^ all are detrimental to successful regeneration.^5,6^ Moreover, soon after SCI, a rim of glial fibrillary acidic protein (GFAP) positive cells is often found to be deposited surrounding the lesion area forming glial scar walls. While GFAP immunoreactivity is often used to label glial scar, glial scar could be composed of many additional cells including oligodendrocytic lineage cells, microglia, macrophages, stromal cells, fibroblasts, leptomengingeal cells, and Schwann cells.^7^ These cells express several inhibitory molecules such as chondroitin sulfate proteoglycans (CSPGs),^7–13^ ephrins,^14,15^ semaphorins,^16–18^ tenascins,^19^ which are thought to be major impediment for axonal regeneration.^20,21^ In recent years the idea of glial scar serving as a physical barrier to block axonal regeneration has been challenged by demonstrations of CNS axons growing through glial scar as long as trophic support was present at the other side of the scar “barrier”.^22–27^ In addition, glial scar is also considered a nature’s effective strategy to prevent the spread of neural inflammation,^28^ which is a major source for secondary lesion and impairment of neural function. That being said, the aforementioned inhibitory molecules in glial scar are still considered negative elements for axonal regeneration. Whether scar removal and when to remove the scar is beneficial or detrimental to SCI repair still remains to be determined.^29–34^

Our group previously reported in a severe acute spinal cord injury models with a removal of 5 mm or 10 mm spinal cord segment in rats or monkeys, respectively, a biodegradable material, chitosan, when loaded with neurotrophin 3 (NT3) allowed for robust *de novo* neural regeneration.^35–37^ The NT3-chitosan biomaterial enabled slow release of this neurotrophic factor into the milieu to create a neurotrophic and anti-inflammatory^38,39^ microenvironment for regeneration over a long period of time, up to 14 weeks.^40^ When NT3-chitosan in a tubular scaffold was inserted into the empty gap in rat or monkey injured spinal cord after spinal cord segment removal, it triggered robust *de novo* regeneration, promoting long-distance axonal growth and engaging endogenous neural stem cells to differentiate into new neurons and forming new neural networks to relay ascending and descending neural signals, leading to sensory and motor functional recovery.^35,36^ These preclinical studies suggested that the biomaterial might be suitable for SCI repair in human. However, when considering clinical trials using NT3-chitosan, the first question is whether one shall target acutely injured patients or during the subacute or chronic phase of SCI. Due to the enormous complexity of SCI, targeting acute phase SCI is quite challenging. It is also difficult to obtain patients’ consent before the long-term outcome of the SCI is relatively clear. Moreover, to obtain statistically meaningful data when targeting acute SCI, larger numbers of patients will be needed because it is difficult to assess the nature and the extent of the injury or to predict the clinical outcome during the acute phase. A significant number of patients might have varying degrees of spontaneous recovery.

To target subacute or chronic phase of SCI appear to be more practical because at such a time point, clinical endpoints of SCI patients become more predictable and patients are also more informed and prepared to make choices of whether or not to be enrolled in trials. Yet, two major issues need to be resolved first: (1) to assess lesion area and progression of secondary lesions for patient selection, (2) to determine whether NT3-chitosan is still effective in treating chronic SCI. To address these two critical issues, we carried out detailed studies using noninvasive magnetic resonance- diffusion tensor imaging (MR-DTI) method to monitor temporal changes in lesion area and/or the scar formation processes, and to determine whether MR-DTI measurements are consistent with anatomical/histological analyses at different times after complete SCI in rat. We found that two types of measurements were relatively consistent. Three months after the initial complete SCI, animals were subjected to a second surgery. MR-DTI imaging was used as a reference to guide the clearance process of the lesion core via vacuum suction of cystic tissues, trimming of solid scar tissues within the lesion core, as well as thinning of glial scar walls, without touching tissues outside the boarder of the lesion core. A soft gel form of NT3-chitosan was then injected into the lesion core after the clearance process, and in another group, tubular form of NT3-chitosan was used with slightly more trimming of the two lesion ends. Using anatomical, behavioral, and electrophysiological analyses, we found NT3-chitosan can still elicit axon growth of the cortical-spinal-tracts (CST) as well as new neurogenesis and functional recovery similar to what was reported for NT3-chitosan to repair acute SCI. In addition, MR-DTI was used to monitor the regeneration process over time, in a noninvasive manner. Taken together, these data support the usage of MR-DTI to map the lesion area in subacute and chronic-SCI phases as well as monitoring the regeneration process. This study laid a foundation for moving NT3-chitosan forward to clinical trials aiming at treating subacute or chronic SCI.

## Results

### 3D-reconstruction of glial scar at different times after complete SCI

**Fig. 1:**
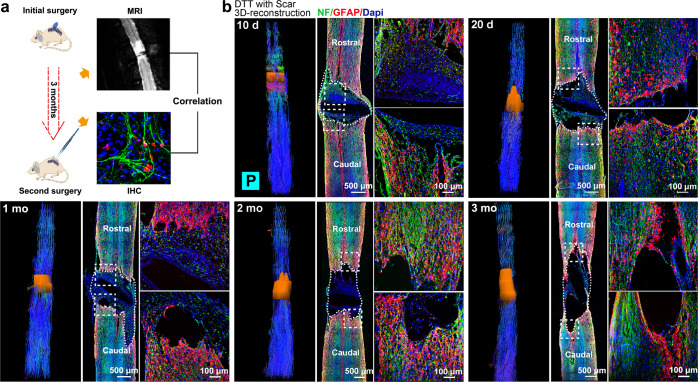
Noninvasive monitoring of lesion progression. **a** Schematic diagram of MRI-DTI imaging to identify lesion area in spinal cord tissue. After the initial operation, a series of MRI-DTI scans were performed at 10 days, 20 days, 1 month, 2 months, and 3 months post-surgery. **b** Diffusion tensor tractography (DTT) images with 3D reconstructed models of lesion area and corresponding immunohistochemistry of the same cord after DTT analyses at different time points post-surgery. P means posterior

Forty-eight adult Wistar female rats weighing about 220 g were subjected to the initial complete transection of the spinal cord (SC) at thoracic level 7–8 (T7–8). Following the transection, a circular plastic film about 0.1 mm thick was placed into the lesion site to completely separate the two ends of the severed spinal cord, preventing any regeneration. Following the initial injury, five rats were subjected to a series of MRI scans at various times up to 3 months (Fig. 1). Diffusion tensor imaging (DTI) datasets were obtained, and fiber tracking images were calculated. Based on diffusion tensor tractography (DTT) results, 3D model of lesion cores at 10 days, 20 days, 1 month, 2 months, or 3 months following SCI was reconstructed for each animal. The same animals, after MR-DTI scan, were sacrificed and subjected to immunohistochemical analyses using antibodies against neurofilament (NF), glial fibrillary acidic protein (GFAP) to demarcate edges of the lesion cores. DAPI was used to label cell nuclei (Fig. 1b). It was interesting that the lesion core initially contained NF^+^ neuronal entities, likely representing damaged neurons, which eventually disappeared. GFAP immunoreactivity, on the other hand, was always excluded from the lesion core, suggesting some elements or factors in the lesion core repelled astrocytes from entering. Within the lesion core, IBA1 positive and CD45 positive microglia and/or infiltrating leukocytes were present (Supplementary Fig. 1). H&E staining of spinal cord sections were also performed showing changes in lesion areas (Supplementary Fig. 2). Both DTT and histological analyses clearly demonstrated gradual expansion of the lesion area over time (Fig. 1b, Supplementary Fig. 1). Three-dimensional (3D) reconstruction of the lesion area surrounded with GFAP^+^ glial scars by DTT, after two-dimensional (2D) projection, was compared to results from immunohistochemical images in Fig. 1b, and revealed good consistency in length, width, and area, especially after 1 month post lesion (Supplementary Table 1). These results demonstrated that the DTT method was suitable and reliable for longitudinally monitoring scar formation over time in a noninvasive manner.

### Clearance of lesion cores and application of NT3-chitosan to aid chronic-SCI repair

**Fig. 2:**
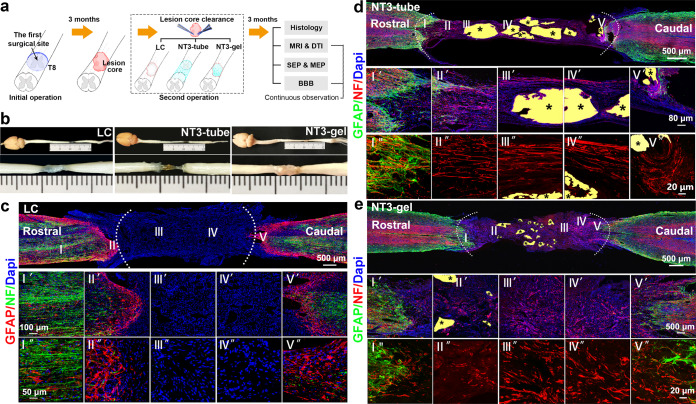
NT3-chitosan promoted endogenous neurogenesis in lesion area after SCI. **a** Schematic diagram of the experimental procedure. **b** Dorsal view of representative spinal cords from three treatment groups: the NT3-tube group, NT3-gel group, and LC group at 3 mo after the second operation. **c**–**e** Immunographs of NF and GFAP-positive cells in the injured/regenerating nerve tissues of LC, NT3-tube, and NT3-gel groups at 3 mo after the second operation. No NF-positive (red) Nerve fibers were found in LC groups (**c**). A mass of neurofilament staining (red) detected in regenerated neural tissues with NT3-tube (**d**) and NT3-gel (**e**) groups. *Labels undegraded chitosan biomaterials. Serially amplified images of highlighted regions

Three months after the initial SCI operation, DTT was used to map the lesion area surrounded by GFAP^+^ glial scars. Animals were subjected to secondary surgeries during which lesion cores were processed by vacuum suction of the cystic tissues and trimming of solid scar tissues within the lesion core with surgical scissors (Fig. 2a). At the anterior and posterior ends of the lesion core, thinning of the scar wall was carefully performed without pocking through the scar wall. Following lesion cores clearance, fifteen animals were equally divided into three groups: (1) no further treatment as lesion controls (LC), (2) lesion area being filled with injectable NT3-chitosan gel (NT3-gel), and (3) insertion of an NT3-chitosan tube (NT3-tube). When necessary, additional trimming of scar tissues was performed to allow for good fitting of the tubular material. Previously, NT3-tube was shown to be efficacious in acute SCI repair in our published study,^35–37^ the NT3-tube group was designed to serve as a good control. Animals, after second operation, were monitored for additional 3 months by MR-DTI before they were sacrificed for structural/histological analyses. Electrophysiological analyses (four of five were randomly selected in each group) on sensory and motor evoked potentials (SEP and MEP) were also performed.

### DTI analyses revealed neural tissue regeneration in progress

**Fig. 3:**
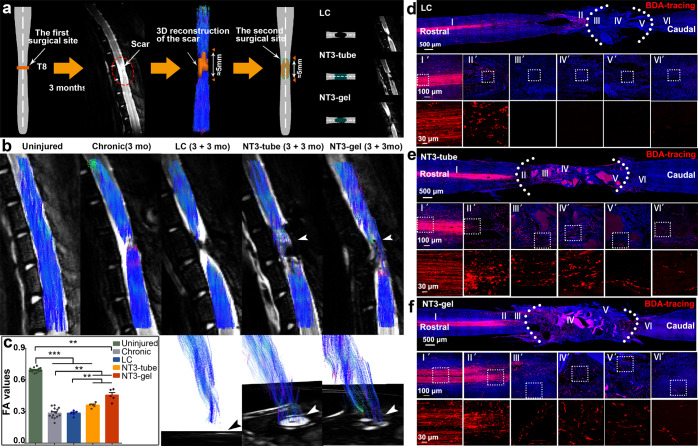
DTI indicating neural tissue regeneration after chronic SCI by NT3-tube and NT3-gel. **a** Diagram illustrating establishment of chronic-SCI models, removal of glial scars, and differences among three different groups (LC, NT3-tube, and NT3-gel). **b** Typical fiber tract reconstructions in Uninjured, Chronic, LC, NT3-tube, and NT3-gel treated spinal cords are displayed. Only NT3-tube and NT3-gel animals demonstrate regenerated fiber bundles (white arrowheads) extending across the surgical site, while LC animals showed no fibers passing through the lesion area. Red lines indicate the region of interest (ROI) of the spinal cord. Lower panel demonstrated DTI fiber tracks over axial images for LC, NT3-tube, and NT3-gel groups. **c** Bar graph of averaged FA values of five groups was displayed. FA values were extracted and calculated from each animal’s ROI. V, ventral; D, dorsal; L, left; R, right; **P < 0.01; ***P < 0.001, by ANOVA (*F* value = 272.39445). Shown are mean ± SEM. Number of animals: uninjured stage, *n* = 15, chronic stage, *n* = 15, and LC, NT3-tube, NT3-gel groups after the second operation, *n* = 5 for each group. See Supplementary Table 2 for exact *P* values. **d**–**f** CST tracking with unilateral cortical BDA injections in LC, NT3-tube, and NT3-gel groups at 3 mo after the second operation

We used DTI and DTT to non-invasively monitor spinal cord injury and regeneration in uninjured (Uninjured), chronically injured (Chronic, 3 months post injury), and LC, NT3-tube, and NT3-gel (3 + 3 months) animals (Fig. 3a). In Uninjured spinal cord, blue colored anterior-posterior (A-P) directional DTI signals filled the whole spinal cord structure in an orderly manner (Fig. 3b). Three months after the first transection, continuous blue A-P signals were interrupted, leaving a gap at the surgical site. Three months after lesion core clearance by the second surgery, DTT showed different results among three groups. In NT3-tube and NT3-gel animals, blue fibular signal bundles reappeared at the surgical site and passed through the initial lesion plane, while LC group showed no signs of reconnection (Fig. 3b). Quantitative comparisons of fractional anisotropy (FA) values among five groups demonstrated the highest FA values in the Uninjured group, and significant improvement elicited by NT3-tube and NT3-gel compared to chronically injured state after first and second operations (Fig. 3c).

Longitudinal studies after the second operation demonstrated that in NT3-tube and NT3-gel groups, at 1 month post second surgery, disrupted blue A-P signal tracks extended into the lesion area and persisted for at least 3 months, but for the LC group, no signals extended into the injury region at any time (Supplementary Fig. 3a). Comparisons of FA values among LC, NT3-tube, and NT3-gel showed that increasing FA values first appeared at the boundary of the surgical site (R2 and C2 level) at 7 days post second surgery, and then gradually extended into the damage center (M level) at 1 month. By 3 months after the second surgery, FA values at all positions in NT3-tube and NT3-gel animals were significantly higher than those in LC groups, indicating that application of NT3-chitosan bioactive and biodegradable material has good efficacies in chronic-SCI repair (Supplementary Fig. 3b).

To demonstrate that DTI results somewhat reflect neural regeneration, we used cortical injection of BDA to label CST, 3 months after second surgery. As shown in Fig. 3d–f, CST axonal regeneration across the lesion area did occur in NT3-tube and NT3-gel groups, whereas no axonal extension into the lesion core was observed with the LC group (Fig. 3d–f).

### Generation of new neurons in lesion area triggered by NT3-chitosan

**Fig. 4:**
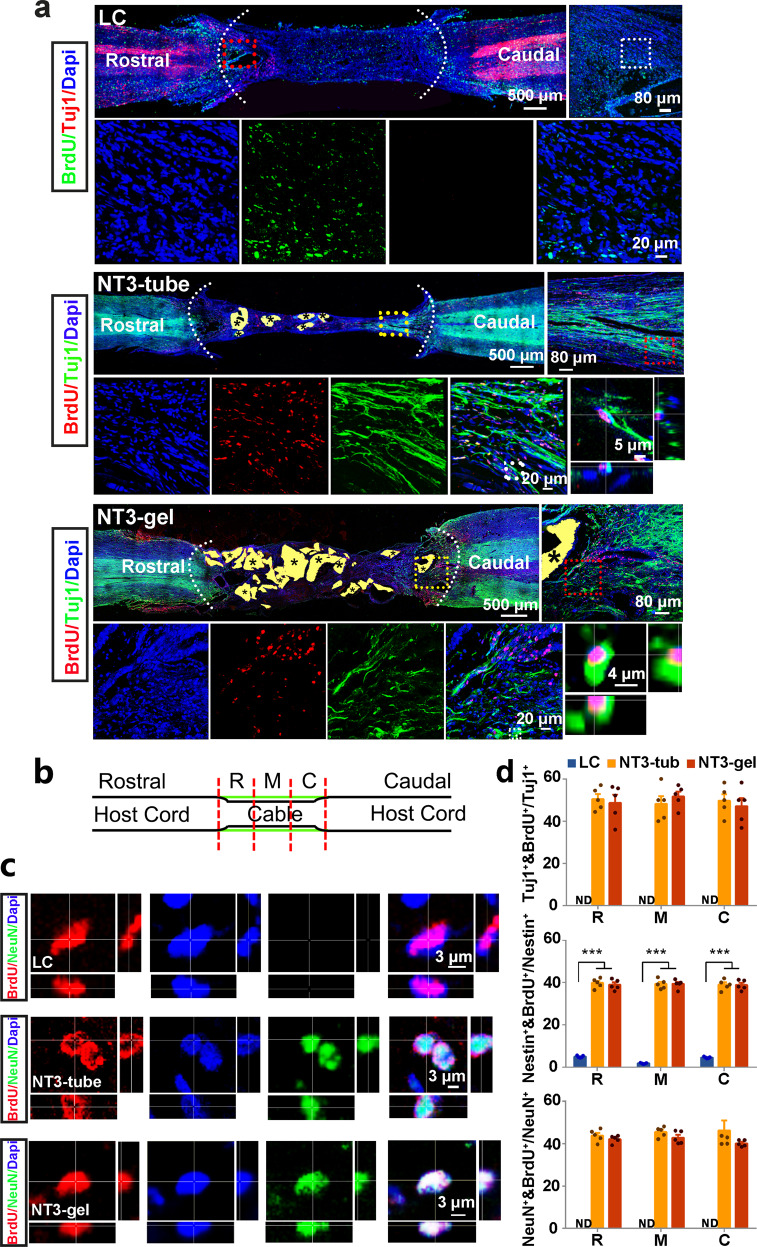
NT3-chitosan promoted new endogenous neurogenesis in lesion area after SCI. **a** Immunographs of BrdU-labeled and Tuj1-positive cells in the regenerating nerve tissues of LC, NT3-tube, and NT3-gel groups at 3 mo after the second operation. No BrdU^+^/Tuj1^+^ cells were found in LC groups. **b** Schematic diagram of the lesion areas examined, which could be divided into three segments, rostral (R), middle (M), and caudal (C) segments. **c** Immunographs of BrdU-labeled and BrdU-positive cells in the regenerating nerve tissues of LC, NT3-tube, and NT3-gel groups at 3 mo after the second operation. No BrdU^+^/BrdU^+^ cells were found in LC groups. *Labels undegraded chitosan biomaterials. Serially amplified images of highlighted regions, as well as single optic sections of confocal images with Z-stack demonstrated co-labeling. **d** Quantification of immunohistochemistry staining of Tuj1^+^ and BrdU^+^ cells showed in (a) and supplementary Fig. 4. ***P < 0.001, by ANOVA or two-tailed independent sample *t* test. Data were presented as mean ± SEM. Number of animals: *n* = 5 for each group. See Supplementary Table 2 for exact *P* values

Anatomical and histological analyses demonstrated that similar to NT3-chitosan repair after acute SCI, in this chronic-SCI model, NT3-chitosan tube also triggered generation of bridging neural tissues containing a large number of Brdu and Tuj1 double-positive newborn neurons (Fig. 4). Brdu was introduced into animals right after the second operation, daily, for 7 days. NT3-chitosan gel, although did not lead to generation of a similar structure at the gross anatomical level as the NT3-chitosan tube, still triggered robust new neurogenesis (Fig. 2b, c). Moreover, those newly born neurons appeared to be generated from Nestin^+^ neural stem/progenitor cells and can further mature into NeuN^+^ neurons (Supplementary Fig. 4**)**. In addition, both NT3-chitosan gel and tube appeared to be anti-inflammatory as they both reduced CD45 and IBA1 immunoreactivities within lesion areas (Supplementary Figs. 5 and 6).

### Protective effect on caudal spinal cord by NT3-chitosan

**Fig. 5:**
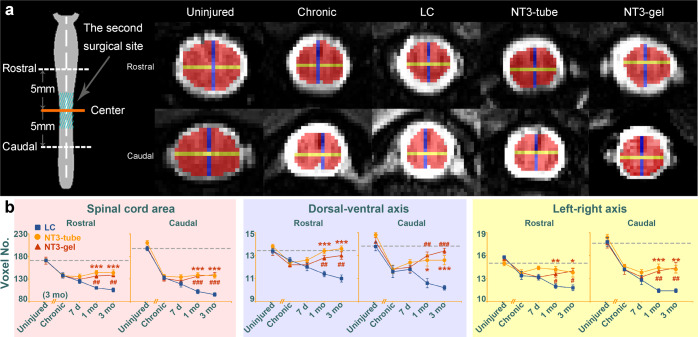
Comparisons of changes in spinal cord atrophy among three groups after the second surgery. **a** Diagram shows sampling points, which are 5 mm away from the center of the lesion core. The lengths of spinal cord dorsal-ventral axis and left-right axis as well as the area at the sampling plane were measured for all sample groups and at different time points. These data are plotted in (**b**) to demonstrate dynamic atrophic changes in spinal cord tissues rostral and caudal to the lesion. *, NT3-tube vs. LC; #, NT3-gel vs. LC; *, ^#^*P* < 0.05; **, ^##^*P* < 0.01; ***, ^###^*P* < 0.001, by ANOVA. Data are presented as mean ± SEM. Number of animals: *n* = 5 for each group. See Supplementary Table 2 for exact *P* values

During spinal cord injury/repair, axonal degeneration/regeneration is a continuous process. To evaluate the protective effect of NT3-tube and NT3-gel on spinal cord caudal to the lesion, MRI measurements were performed (Fig. 5a). Longitudinal investigations demonstrated gradual recoveries in caudal spinal cord volumes and diameters in NT3-tube and NT3-gel groups. The LC group, however, displayed a more pronounced atrophic process in the caudal spinal cord at 1 month and 3 months post second surgery (Fig. 5b).

To address whether second surgical procedure may exacerbate atrophy of spinal cord rostral and/or caudal to lesion area, we monitored animals without second surgery at 3 months and onward till 6 months post first injury as well as the LC group. DATA indicated a trend of worse atrophic progression with the LC group, but the differences did not reach statistical significance (Supplementary Fig. 7a). Importantly, by 6 months, the NT3-gel and NT3-tube group demonstrated recovery of spinal atrophy and were significantly better than animals without 2nd operation (Supplementary Fig. 7b). These data indicated that implementation of NT3-chitosan was beneficial, even though a second operation was involved.

### NT3-chitosan triggered functional recovery after chronic SCI

**Fig. 6:**
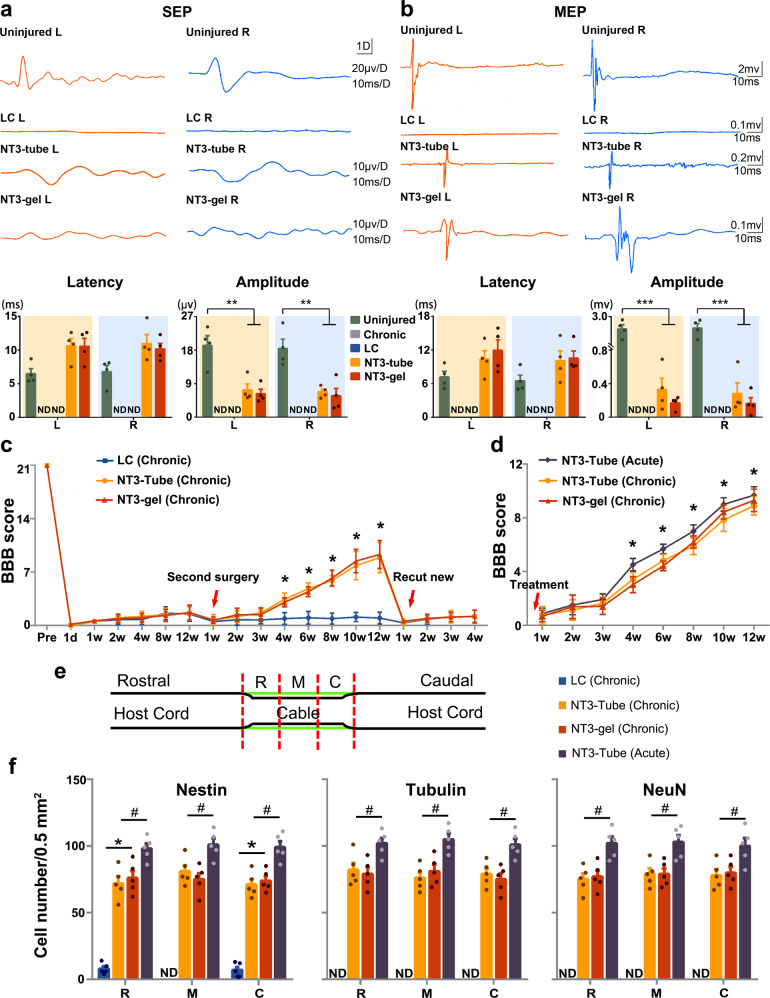
SEP, MEP, and BBB scoring demonstrating functional recovery with NT3-chitosan treatment in chronic SCI after scar removal. **a** and **b** SEPs detected in left primary somatosensory cortex (S1, red) when right anterior tibial muscle was stimulated, and in right somatosensory cortex (green) when left hindlimb muscle was stimulated 3 months after second surgery. SEP is completely abolished in LC group. SEP reappeared in both NT3-chitosan treatment groups. Stimulation of right motor cortex evoked MEPs in left anterior tibial muscles (red), and stimulation of the left motor cortex evoked MEPs in the right hindlimb muscles (green). Two forms of NT3-chitosan biomaterial both allowed partial recovery of MEPs. Statistical analyses of latency and amplitude for the aforementioned experiments were shown. L, left side, R, right side, *P < 0.05, **P < 0.01, ***P < 0.001, by ANOVA (detailed *F* values: SEP Latency: L, 5.232251 and R, 4.327416; SEP Amplitude: L, 14.572455 and R, 13.909387. MEP Latency: L, 1.447755 and R, 3.710467; MEP Amplitude: L, 40.295106 and R, 40.348461). Data are presented as mean ± SEM. Number of animals: *n* = 4 for each group. ND, not determined. **c** BBB open-field walking scores of bilateral hindlimbs for the NT3-tube, the NT3-gel, and the LC groups over time, up to 3 months after the second operation. At 4 weeks after the second operation and thereafter, the NT3-tube group and the NT3-gel group showed significantly higher BBB scores compared with the LC group. After the regenerated tissue resection, the BBB scores for NT3-tube group and the NT3-gel group decreased to the level of the LC group. *P < 0.05, by ANOVA; Data are presented as mean ± SD. Number of animals: *n* = 8 for each group. **d** The NT3-tube (Chronic) group and the NT3-gel (Chronic) group had statistically significant slightly lower BBB scores as compared to the NT3-tube (Acute) group at 4–12 weeks post operation. *P < 0.05, by ANOVA; Data are presented as mean ± SD. Number of animals: *n* = 8 for each group. See Supplementary Table 2 for exact *P* values. **e** Schematic diagram of the lesion areas examined, which could be divided into three segments, rostral (R), middle (M), and caudal (C) segments. **f** Quantification of immunohistochemistry staining of nestin^+^, NeuN^+^, and Tuj1^+^ cells shown in Fig. 4a and S4. Data were presented as mean ± SEM; *n* = 5 for each group; *, ^#^*P* < 0.05, by ANOVA; the NT3-tube (Acute) group is used to compared with the other three groups. See Supplementary Table 2 for exact *P* values

We used electrophysiological analyses to assess the functional outcome following NT3-chitosan treatment. Somatosensory evoked potentials (SEP) and motor evoked potentials (MEP) exhibited stable wave forms in uninjured spinal cord (Fig. 6a, b). 3 months after the second operation, no SEP or MEP signals were detected in LC rats, whereas significant gains of both SEP and MEP signals were detected in both NT3-tube and NT3-gel treated animals, although latencies were still longer and amplitude smaller than those in uninjured controls (Fig. 6a, b), indicative of partial recovery.

Basso–Beattie–Bresnahan (BBB) open-field walking scale was also used to measure hindlimb locomotor activity of 24 rats after SCI and functional recovery triggered by NT3-chitosan. BBB scoring was performed double blindly to assure objectivity. Data demonstrated that both NT3-gel and NT3-tube groups acquired better locomotor skills after chronic SCI (Fig. 6c). Small but statistically significant differences were observed between NT3-chitosan-treated chronic SCI versus NT3-chitosan-treated acute SCI animals (Fig. 6d). These data suggested that chronic SCI is more difficult to treat or perhaps longer time is needed.

We also compared the extent of NT3-chitosan triggered neurogenesis in this chronic-SCI model to that in the acute SCI model, and found that 3 months after NT3-chitosan application, the numbers of Nestin^+^ NPCs and the numbers of Tuj1^+^ or NeuN^+^ neurons in the lesion area, in the case of chronic SCI, is about 20% less than that in acute SCI model (Fig. 6e, f, Supplementary Fig. 4), again suggesting that chronic SCI is more difficult to repair, or perhaps, it would take a longer time to repair. On the other hand, less neurons generated in chronic-SCI repair might, at least in part, account for less functional recovery, compared to acute SCI repair.

### Trimming of solid scar tissues appeared to be necessary for chronic-SCI repair

**Fig. 7:**
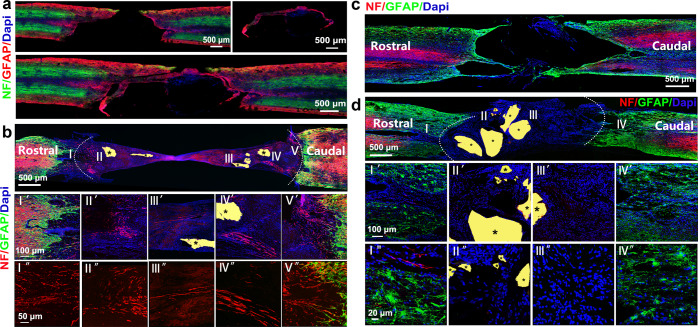
Improving the microenvironment in the damaged area can promote nerve regeneration. **a** Immunographs of NF and GFAP-positive nerve fibers during the second operation immediately. The small image on the right shows the scar tissue that has been cut off. The following diagram shows a reconstruction of the scar back to the cord. **b** Immunographs of NF and GFAP-positive nerve fibers in the regenerating nerve tissues of groups at 3 mo after the second operation. A mass of neurofilament staining (red) detected in regenerated neural tissues. **c** Immunographs of NF and GFAP-positive nerve fibers in chronic-SCI rats before aspiration of the cystic tissues at 3 mo post the first operation. **d** Immunographs of NF and GFAP-positive nerve fibers in aspiration-only rats with materials treatment at 3 mo after the second operation. No NF-positive (red) nerve fibers were found in this group. *Labels undegraded chitosan biomaterials. Serially amplified images of highlighted regions

Since it has been reported that glial scar not only did not serve as absolute physical barriers to inhibit axonal regeneration, but might even aid SCI repair,^27^ we decided to address whether trimming of solid scar tissues within the lesion core and/or thinning of GFAP+ glial scar wall at rostral and causal ends of the lesion core is necessary for chronic-SCI repair. We performed only suction of cystic tissue within the lesion core without scar trimming, followed by NT3-gel injection. To our surprise, 3 months later, we observed little, if any, regeneration (Fig. 7). Anatomical results demonstrated that without scar trimming, it was difficult to allow injected NT3-chitosan to fill the entire lesion core. As such, non-neural inflammatory cells re-occupied the lesion core at places where NT3-chitosan was not present. Interestingly, even at places where NT3-chitosan was present, neural regeneration still failed to occur, suggesting that inhibitory signals from infiltrating inflammatory non-neural cells, or excessive scar tissues are detrimental to neural regeneration.

## Discussion

To date, effective treatment for SCI, including subacute or chronic SCI, has not been developed. Through current study, we demonstrated feasibility of using noninvasive MR-DTI and DTT to monitor the process of scar formation at anatomical levels. Such information can also be used to aid clinical trial designs for patient enrollment to assure similar degrees of damage amongst trialed patients. Moreover, DTI and DTT imaging can be used to guide the surgical clearance of the lesion core to create spaces for introducing the neural regeneration promoting NT3-chitosan biomaterial to elicit powerful regeneration processes involving not only axonal regeneration but also new neurogenesis and likely formation of new relay neural networks to resume interrupted neural transmissions in subacute or chronic phase of SCI.

In our previous study, we demonstrated that NT3-chitosan elicited repair of acute complete SCI,^35–37^ although the complete transection plus spinal cord segment removal was a very severe injury where no spontaneous regeneration was possible, some concerns were raised regarding the clinical relevance of the model, because in the clinics such an extreme traumatic SCI almost never happened. Whether our previous study is translatable to human clinical settings was questioned. In this current study, we found that although the initial complete transection of the spinal cord was a clean cut, and no spontaneous regeneration was possible by insertion of a plastic film, the lesion area became irregular three months later, possibly exacerbated by inflammatory responses triggered by the plastic film as a foreign object. Being able to map out the lesion area, which serves as a reference for subsequent clearance of the lesion core without additional injuries to healthy spinal cord tissues, could be important in clinical settings regardless of the initial form of traumatic SCI.

Although in recent years, several studies suggested that perhaps glial scar did not present an absolute impediment to axonal regeneration,^27^ therefore might not need to be removed, the inflammatory tissues in the lesion core still likely need to be removed for successful regeneration. In addition, clearance of the lesion core also created space for NT3-chitosan bio-effective material to be introduced into the injured spinal cord to elicit regeneration. Interestingly our study demonstrated that only extracting away the cystic inflammatory tissues without trimming away solid scar tissue followed by NT3-chitosan application failed to elicit regeneration. On the other hand, trimming and thinning of the glial scar walls allowed NT3-chitosan to trigger de-novo neural regeneration, demonstrating that the glial scar is, indeed, a barrier against regeneration. Consistent with our results, a recent immunohistochemical study with SCI patients proved that amyloid precursor protein (APP) and oxidized phospholipids within the surrounding rim, but not the lesion core, remained profoundly high level for ongoing neuronal damage up to months/years post-SCI.^41^ Our findings, together with this prior study, suggest that glial scar should be removed as much as possible to gain better recovery. This is encouraging because this means operation could be executed without risking harming of the potentially healthy spinal cord tissues.

In acute SCI study, newly born neurons further matured in the presence of NT3-chitosan and formed nascent functional synaptic networks in the lesion area, which signified a unique regeneration strategy by engaging endogenous NSCs.^35^ In the current chronic-SCI study, NT3-chitosan also enabled generation of neural tissue bridges, which contained a large number of Brdu and Tuj1 double-positive newborn neurons. Similar to our previous acute SCI study, newly born neurons in this chronic-SCI study appeared to be generated from Nestin+ neural stem/progenitor cells and matured into NeuN+ neurons. Obvious recoveries of electrophysiological signals (SEP and MEP) were also observed in NT3-chitosan-treated animals. Based on these results, it is likely that, in chronic-SCI models, NT3-chitosan could also induce newly born neurons to further mature with extensive outgrowth of axons and dendrites and further participate in the formation of new relay circuitries. Our previous back-to-back studies with NT3-chitosan in acute SCI rats^35,39^ had discussed the mechanisms underlying the NT3-Chitosan-induced regeneration by using weighted gene co-expression network analysis, which revealed that, by gene module expression, enhanced new neurogenesis and angiogenesis, and reduced inflammatory responses were keys to confer the effect of NT3-chitosan on spinal cord regeneration. In the current study, promotion of axonal regrowth and reduction of inflammation in either NT3-chitosan tube or gel group were also observed, suggesting that spinal cord regeneration strategies in chronic stage, after clearance of the lesion scar, were likely similar to that in acute stage.

In this study, we used NT3-chitosan tube as a positive control and for better comparison to our previously published NT3-chitosan repair of acute SCI work.^35,36^ Obviously unlike NT3-chitosan gel, which can be adapted to any shapes of the lesion core after clearance of the lesion core, to insert an NT3-chitosan tube, some minimal damage of the potentially healthy spinal cord tissue may be inevitable. Fortunately, for most functional outcome measurements, NT3-chitosan gel appeared to be as effective as NT3-chitosan tube. We believe that NT3-chitosan gel might be more suitable for clinical usage than NT3-chitosan tube, because any damage of potentially healthy spinal cord tissue during treatment should be avoided at all cost.

Comparing NT3-chitosan tube treatment of chronic SCI versus acute SCI, it is obvious that chronic SCI is more difficult to treat or perhaps longer time is required. The slightly less robust recovery on BBB scoring comparing the chronic to the acute model is also correlated well with slightly less potent new neurogenesis between the two models, consistent with the notion that new neurogenesis is at least in part underlying the mechanisms by which NT3-chitosan triggered neural repair after SCI. Moreover, another difference between acute versus chronic SCI is atrophy of the distal spinal cord, which might also contribute to increased difficulty in repair. Our MRI data demonstrated that 3 months after the second operation with NT3-chitosan, atrophy of the caudal spinal cord was partially reversed. It remains to be determined whether caudal spinal cord atrophy can be completely reversed back to that of uninjured spinal cord with longer recovery time. Regardless of the answer, such a type of atrophy might be associated with slightly reduced efficacy of NT3-chitosan treating chronic SCI versus acute SCI.

Another concern is the impact of gender selection in this study. The gender bias has been reported in clinics as SCI is more common in men,^42,43^ but a consistent conclusion of the effect of sex differences on SCI is still lacking. Datto et al.^44^ reported that female rats demonstrated improved locomotor recovery and greater preservation of spinal cord parenchyma after SCI compared to males. A recent age-matched functional and histological gender comparison in thoracic contused rats, however, demonstrated similar lesion volume, spared myelin, glial reactivity, and BBB scores between genders.^45^ Scivoletto et al.^46^ also showed same neurological and functional recoveries between the two genders in the clinically matched cohorts. Since female rats have a shorter urethra, they are easier to be cared for daily, after surgery. For this reason, female rats were chosen in the present study. It is speculated that gender differences might not have an influence on the findings of this study, but an across-gender experiment is still needed to provide definitive evidences.

Taken together, this study addressed some of the key questions for pushing NT3-chitosan into the next phase of clinical studies. Although rat chronic-SCI models were used here, the noninvasive MR-DTI and DTT method mapping and clearance of lesion scars, as well as development of injectable NT3-chitosan are all highly translatable to human clinical settings. Based on our previous success in using NT3-chitosan to repair acute SCI in rats and monkeys, it is likely that this NT3-chitosan material, after proven safe, should be ready for clinical trials to treat subacute and chronic SCI.

## Methods and materials

### Preparation of the NT3-chitosan tube

In a modified method,^35^ under sterile conditions, 2% (wt/vol) solution of poly-N-acetyl glucosamine derived from 85% (wt/vol) deamidized chitosan (Sigma-Aldrich) in 100 mL of water containing 2% (wt/vol) acetic acid was plasticized by treatment with 1 g of di(hydroxyethyl) sulfoxide, which had a melting point of 112–113 °C, and 1 g of lithium chloride. This mixture was thoroughly stirred. A 2.0-mm-diameter glass capillary was washed, sterilized at high pressure, dried, vertically immersed in the foregoing chitosan solution, withdrawn slowly, and dried while keeping the tube vertical. This process was repeated until the inner and outer diameters reached 2.0 mm and 2.2 mm, respectively. The dried glass capillary with the chitosan tube was immersed in NaOH solution for 1 h, and then in distilled water. The distilled water was changed frequently until it became nonalkaline. The glass capillary was discarded, leaving a transparent chitosan tube. The tube was cut into a specific length (based on the MRI results), immersed in 75% (vol/vol) alcohol for sterilization, and washed with PBS.

NT3-chitosan carriers were prepared according to a modified published method.^40,47^ Under sterile conditions, 10 mg of 85% deacetylated chitosan particles (Sigma-Aldrich) were dissolved in 10 mL of sterile deionized water at pH 7.2, allowed to swell for 6 h, and then centrifuged. The supernatant was then discarded. The swollen chitosan particles were frozen at −20 °C for 24 h, and then at 4 °C for 10 h. NT3 (Sigma-Aldrich) was reconstituted to 100 μg/mL in sterile cold deionized water, and 100 ng of NT3 was mixed with chitosan particles in solution at 4 °C. After stirring at 4 °C for 6 h, NT3-loaded chitosan carrier mixture was vacuum-cooled and dried. The dried chitosan particles loaded with NT3 were added to a type I collagen solution, stirred for 30 min, centrifuged, collected, and stored at 4 °C. Then the chitosan carriers (2 mg per 1 mm tube) loaded with NT3 (10 ng per 1 mg carriers) was injected into the middle part of the chitosan tube and kept at 4 °C.

### Preparation of the NT3-chitosan gel

NT3-chitosan gel was prepared similar to the aforementioned NT3-chitosan carrier mix.^40,47^ Under sterile conditions, 10 mg of 85% deacetylated chitosan particles (Sigma-Aldrich) were dissolved in 10 mL of sterile deionized water at pH 7.2, allowed to swell for 6 h, and then centrifuged. The supernatant was then discarded. The swollen chitosan particles were frozen at −20 °C for 24 h, and then at 4 °C for 10 h. NT3 (Sigma-Aldrich) was reconstituted to 100 μg/mL in sterile cold deionized water, and 100 ng of NT3 was mixed with the chitosan particles in solution at 4 °C. After stirring at 4 °C for 6 h, the NT3-loaded chitosan gel mixture was vacuum-cooled and dried. The dried chitosan particles loaded with NT3 were added to a type I collagen solution, stirred for 30 min, centrifuged, collected, and stored at 4 °C.

### Animal models

Forty-eight adult female Wistar rats weighing 200–220 g was used in this study. In each experiment, numbers of animals were chosen to satisfy the statistical test requirements. Five rats were used for monitoring the lesion progression. Four rats were used to verify the effect of scar tissue clearance. The rest 39 rats (15 for MR-DTI, electrophysiology, and anatomy; 24 for BBB assessments) were equally divided into three groups after surgery: lesion control, NT3-chitosan tube and NT3-chitosan gel groups and none of them were lost in surgery or experiments. Complete randomization was applied for group assignment and for experimental selection.

The rats were anesthetized using intraperitoneal injections of Equithesin (3 ml/kg; Sigma-Aldrich). To prepare a thoracic spinal cord transected rat model, we performed laminectomy under an operating microscope, followed by a complete transection at thoracic level 7–8 (T7–8). After complete transection, a piece of plastic film was inserted into the lesion site to ensure complete disconnection of the spinal cord tissue. Subsequently, the skin was stapled and the rats were kept for recovery. After three months, some rats were subjected to second surgery. First, plastic films and glial scars were processed based on MRI results, and that scar tissues have a higher density and tougher than normal spinal cord tissue. Animals were divided into three groups. In the NT3-chitosan tube (NT3-tube) group, chitosan tube seeded with the NT3-chitosan carrier was implanted into the lesion area after additional transections at the rostral most and caudal most positions of the scar. The NT3-chitosan gel (NT3-gel) group, received injections of NT3-chitosan gel into the irregular space after scar removal. The LC group received no treatment after scar clearance. Muscles and skin were closed in layers. After the second operation, the rats were kept warm and placed on beds of sawdust. The rat bladders were massaged three or four times daily, and intramuscular injections of ampicillin were administered (50 mg once daily up to 1 wk after the operation) to prevent infections. All animals that have undergone secondary surgery received an i.p. injection of BrdU (50 mg/kg body weight, in 0.9% NaCl solution Sigma-Aldrich) twice daily for continuous 7 days.

All experimental procedures were performed in accordance with the standard of Experimental Animal Center of Capital Medical University and Beijing Experimental Animal Association (IACUC: No. AEEI-2017–033).

### MRI and DTI

MRI and DTI of all animals (*n* = 15) were accomplished using a 7.0 T MRI scanner (Bruker BioSpec, Karlsruhe, Germany) before SCI, 3 months after the first operation, and at 7 days, 1 month and 3 months after the second operation (*n* = 5, three groups). Anatomical scans were conducted with the following parameters: rapid acquisition with relaxation enhancement (RARE) sequence, 20 slices, slice thickness = 1 mm with no gap, 256 × 256 matrix, FOV = 4 cm × 4 cm, TR = 3000 ms, TE = 45 ms, NEX = 6. Single-shot spin-echo echo-planar imaging (SE-EPI) was used for spinal DTI sequence. Axial-orientation diffusion-weighted (DW) images were acquired using a Single-shot spin-echo echo-planar imaging (SE-EPI) sequence and had the same centering as axial anatomical images. Acquisition parameters were: 20 slices (without gap), 128 × 128 matrix, FOV = 1.88 × 1.88, TR = 5000 ms, TE = 23 ms, voxel size = 0.147 × 0.147 × 1 mm^3^, *b* value = 0 and 670 s/mm^2^. Diffusion encoding was in 30 non-colinear gradient directions. Saturation bands were set on rat’s chest and abdomen to reduce movement artifacts. MRI matched cardiac gating was used during the whole scanning. Warm air was forced into the magnet bore to maintain the animal body temperature.

### Three-dimensional (3D) reconstruction of the scar

In order to determine the main diffusion direction of each voxel in white matter of lesion area, RGB values of these voxels in color-FA maps were extracted. When the color of the voxel is dominated by the blue component (i.e. the *B* value is the largest), the main diffusion direction of the voxel is along with the superior-inferior orientation.^48,49^ Otherwise, the main diffusion direction of the voxel is perpendicular to the cord. We defined voxels which main diffusion direction is perpendicular to the cord and without the blue fiber bundles crossing as the scar voxels. Based on this method, scar area in each slice was drawn to reconstruct the three-dimensional scar model. To validate that this noninvasive method could be used to monitor scar formation, five rats were selected for MRI scans at 10 days, 20 days, one month, two months, and three months after the first surgery, and scar reconstruction results were found to be consistent with corresponding pathological results. The matching rate between results of the scar reconstruction (SR) and the immunohistochemistry (IHC) is calculated by the following formula:

$${\rm{Matching}}\;{\rm{rate}} = \left( {1 - \frac{{|{\rm{IHC}} - {\rm{SR}}|}}{{\rm{IHC}}}} \right) \times 100{{{\mathrm{\% }}}}$$

Where matching rate is between 0% and 100%, the better consistency between the scar reconstruction and the immunohistochemistry results, the larger matching rate values.

### DTI data processing and distal spinal atrophy quantification

DTI data were processed and analyzed through the MedINRIA software (http://www-sop.inria.fr/asclepios/software/MedINRIA/). The tractography algorithm was based on tri-linear log-Euclidean interpolation and was fully multithreaded, so no seed points were required.^50,51^ We set the entire spinal cord area as the region of interest (ROI) manually in anatomical images (Fig. 3b, middle plane). Special care was taken to avoid the empty spaces and the partial volume effect of cerebrospinal fluid in ROI selection.

To quantitatively assess the spinal atrophy, we measured the cross-sectional area and the two diameters (sagittal diameter and transverse diameter) at the distal end of the damaged site (Fig. 5a). All quantified values were obtained from spinal cord axial anatomical images. To avoid the cerebrospinal fluid and other areas, all contours of the spinal cord are manually outlined. The number of voxels in the spinal cord is the quantification value of the cross-sectional area. The maximum number of voxels of the dorsal-ventral and left-right orientation are the quantification value of the sagittal diameter and transverse diameter, respectively.

### BrdU injection

To label proliferating cells and newborn neurons, all rats from three groups received an i.p. injection of BrdU (50 mg/kg body weight, in 0.9% NaCl solution, Sigma-Aldrich) immediately after the second operation twice daily for 7 days. Animals were then sacrificed 3 months after operation.

### Immunohistochemistry/Fluorescence staining

The primary antibodies included rabbit polyclonal anti-nestin (1:100; Abcam ab93157) to label neural stem cells, rabbit monoclonal anti-βIII-tubulin (Tuj1, 1:400; Sigma T2200) to label immature neurons, rabbit monoclonal anti-NeuN (1:500; Abcam ab177487) to label mature neurons, mouse monoclonal anti-5-bromo-2-deoxyuridine (BrdU, 1:200; ZM-0013), rabbit polyclonal anti-glial fibrillary acidic protein (GFAP, 1:300; Zymed), mouse anti-Neurofilament (NF, 1:200; Zymed), rabbit monoclonal anti-CD45 (CD45, diluted 1:100, Millpore), rabbit monoclonal anti-IBA1(1:300, Wako), mouse monoclonal anti-GFAP (1:400, Millpore) are used in the study. The spinal cord tissue including the lesion area was embedded in OCT compound (Sakura Finetek) and sliced longitudinally with a cryostat microtome to obtain 10 μm thick sections. The sections were washed three times with 0.01 M PBS and then incubated with the primary antibodies at 4 °C overnight and washed with 0.01 M PBS three times. Alternatively, after primary antibody incubation, the sections were incubated with appropriate secondary antibodies conjugated to various fluorescent labels, such as Texas red-conjugated Affinipure goat antimouse IgG and CyTm2-conjugated Affinipure goat anti-rabbit IgG (Jackson Laboratory, diluted 1:300), at room temperature for 3 h in the dark. The sections were covered with coverslips and Vectashield mounting medium containing DAPI (Vector Laboratories), and examined under a confocal microscope (TCS SP8, Leica, Germany). Morphological analysis and quantification were performed as described previously.^35^ Briefly, to quantify the number of Nestin^+^, Tuj1^+^, and NeuN^+^ cells in the damaged zone (LC) or regenerative tissue (NT3-chitosan), longitudinal sections including the lesion area were examined with an Olympus Image System (Olympus, Demark). Ten to fifteen longitudinal sections were selected based on the odd- or even-number set with 50 μm intervals via serial sectioning. The numbers of positively immune-labeled cells expressing various markers were counted manually in the 0.5 mm^2^ areas in the rostral, middle, and caudal segments of the damaged zone or regenerative tissue through a whole series of selected sections. Positive cells were expressed as averaged number/0.5 mm^2^. To identify the double-labeled cells, a confocal microscope (TCS SP8, Leica, Germany) with sequential mode were used. Ten longitudinal sections (10 μm/section) spaced 50 μm apart were performed. Each BrdU-positive cell in high magnification was manually examined in its full z dimension, and only those cells that the BrdU-positive nucleus was unambiguously associated with a given marker were considered double-labeled. The numbers of cells expressing various markers were then counted manually using the same method as described above.

### Electrophysiological studies

Electrophysiological assays were performed as described previously.^52,53^ Indices measured included SEP and MEP. The latency and amplitude measurements with stimulation at 10 mA for MEP and 3 mA for SEP were recorded for statistical analysis (*n* = 4 for each group). SEP and MEP parameters of normal rats were used as normal controls. Three months after the first SCI operation, electrophysiological tests were performed on all experimental rats. Only one that showed no SEP or MEP signals were used as chronically injured animals for subsequent study. Rats with detectable SEP and/or MEP would be considered incompletely injured and excluded from the subsequent study.

### Behavioral assessment

Before the first operation, and at day one and each week following the first and the second operation, observers who were blinded to the treatment methods and groups performed BBB scoring in an open field, to evaluate restoration of hindlimb locomotor function after SCI.^35,54,55^

### Statistical analyses

For MRI analysis, the one-way analysis of variance (ANOVA) with the Bonferroni test (Homogeneity of variance) or Dunnett’s T3 test (Inhomogeneity of variance) was used to compare MRI data including FA values and quantitative value of spinal cord atrophy among different groups. The one-sample Kolmogorov-Smirnov test was used for data normality analysis. All data were normally distributed. *P* < 0.05 was considered statistically significant. All values are presented as mean ± SEM.

For other analysis, unless stated otherwise, data are presented as mean ± SEM. The Shapiro-Wilk test was used for data normality analysis. All data were normally distributed. Levene’s test was used to test for homogeneity of variance. One-way ANOVA with the Bonferroni test (Homogeneity of variance) or Dunnett’s T3 test (Inhomogeneity of variance) or two-tailed Independent Sample T Test was used to determine statistical differences except BBBs results (two-way repeated-measures ANOVA). *P* < 0.05 was considered to indicate a statistically significant difference.

## Supplementary information

Supplementary information

## Data Availability

Any data associated with this study are available from the corresponding author upon reasonable request.
