# Supplementary material for: Chronic spinal cord injury repair by NT3-chitosan only occurs after clearance of the lesion scar
Source: Signal Transduct Target Ther. 2022 Jun 17;7:184. doi: 10.1038/s41392-022-01010-1 (PMC9203793; doi:10.1038/s41392-022-01010-1)
Supplement: Supplementary file 1 — Supplementary information [file 41392_2022_1010_MOESM1_ESM.pdf]

Supplementary information

Chronic spinal cord injury repair by NT3-chitosan only occurs after clearance of the lesion scar

Can Zhao[1,2#], Jia-Sheng Rao[1#], Hongmei Duan[3#], Peng Hao[3#], Junkui Shang[3], Wen Zhao[3], Yudan Gao[3], Zhaoyang Yang[3]*, Yi Eve Sun[4,5]*, Xiaoguang Li[3,1]*

*Corresponding authors.

E-mail: wack_lily@163.com, yi.eve.sun@gmail.com, or lxgchina@sina.com

This PDF file includes:

Supplementary Figures 1 to 7

Supplementary Table 1, 2

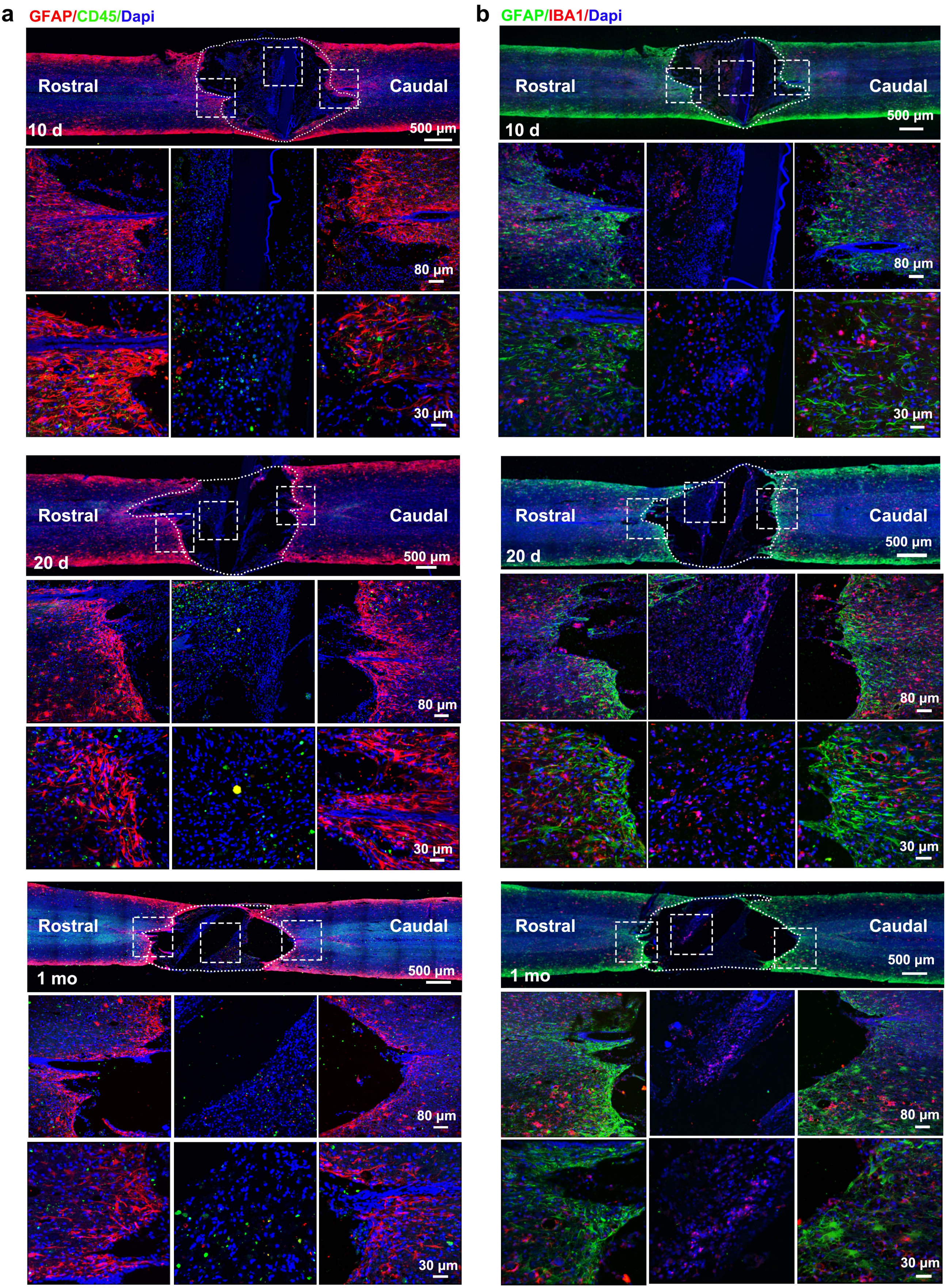
a
GFAP/CD45/Dapi
Rostral
Caudal
10 d
500 µm
80 µm
30 µm
Rostral
Caudal
20 d
500 µm
80 µm
30 µm
Rostral
Caudal
1 mo
500 µm
80 µm
30 µm
b
GFAP/IBA1/Dapi
Rostral
Caudal
10 d
500 µm
80 µm
30 µm
Rostral
Caudal
20 d
500 µm
80 µm
30 µm
Rostral
Caudal
1 mo
500 µm
80 µm
30 µm

SI Figure Legends

Fig. S1. Immunofluorescent staining of inflammatory cells following the initial spinal cord injury. (a) Immunofluorescent staining of GFAP and CD45 at different times post initial surgery. Note the presence of CD45-positive immune cells in the lesion core, while GFAP-positive cells were localized only at the periphery of the lesion area. (b) Immunofluorescent staining of GFAP and IBA1 at different times after operation. IBA1-positive microglia were found in the lesion core, whereas GFAP-positive cells were only found at the periphery of the lesion area.

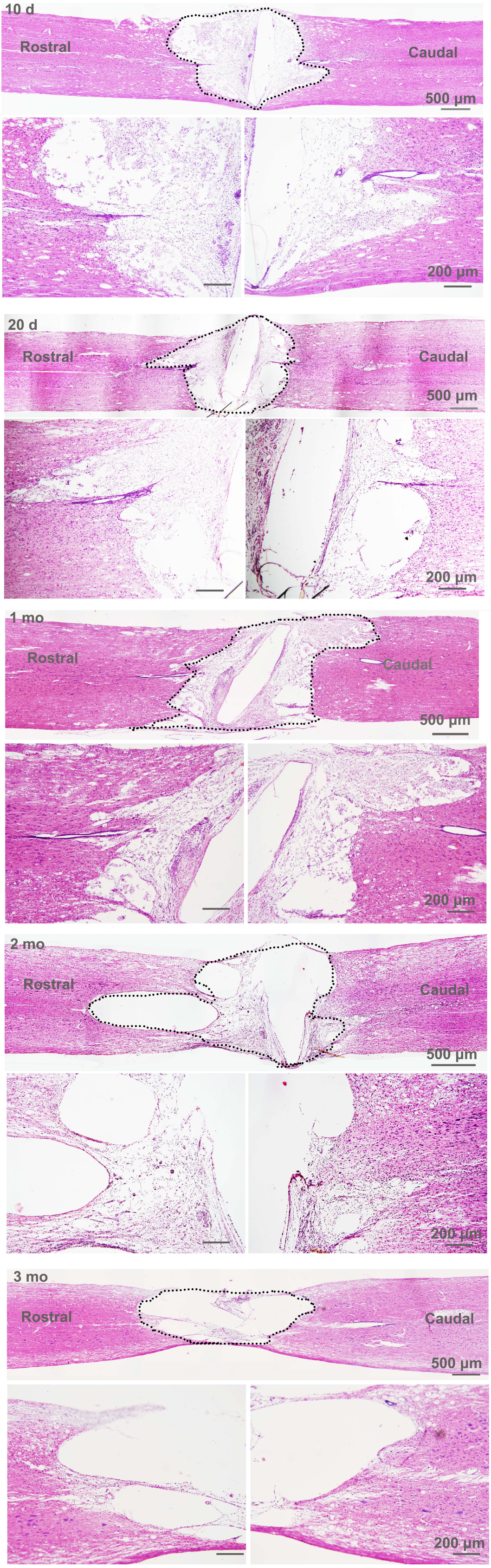

10 d
Rostral
Caudal
500 μm
200 μm
20 d
Rostral
Caudal
500 μm
200 μm
1 mo
Rostral
Caudal
500 μm
200 μm
2 mo
Rostral
Caudal
500 μm
200 μm
3 mo
Rostral
Caudal
500 μm
200 μm

Fig. S2. HE staining of spinal cord tissue at different time points after the initial/primary operation. Over time post initial complete transection of the spinal cord, the area of damaged tissue demarcated by scar tissues gradually increased.

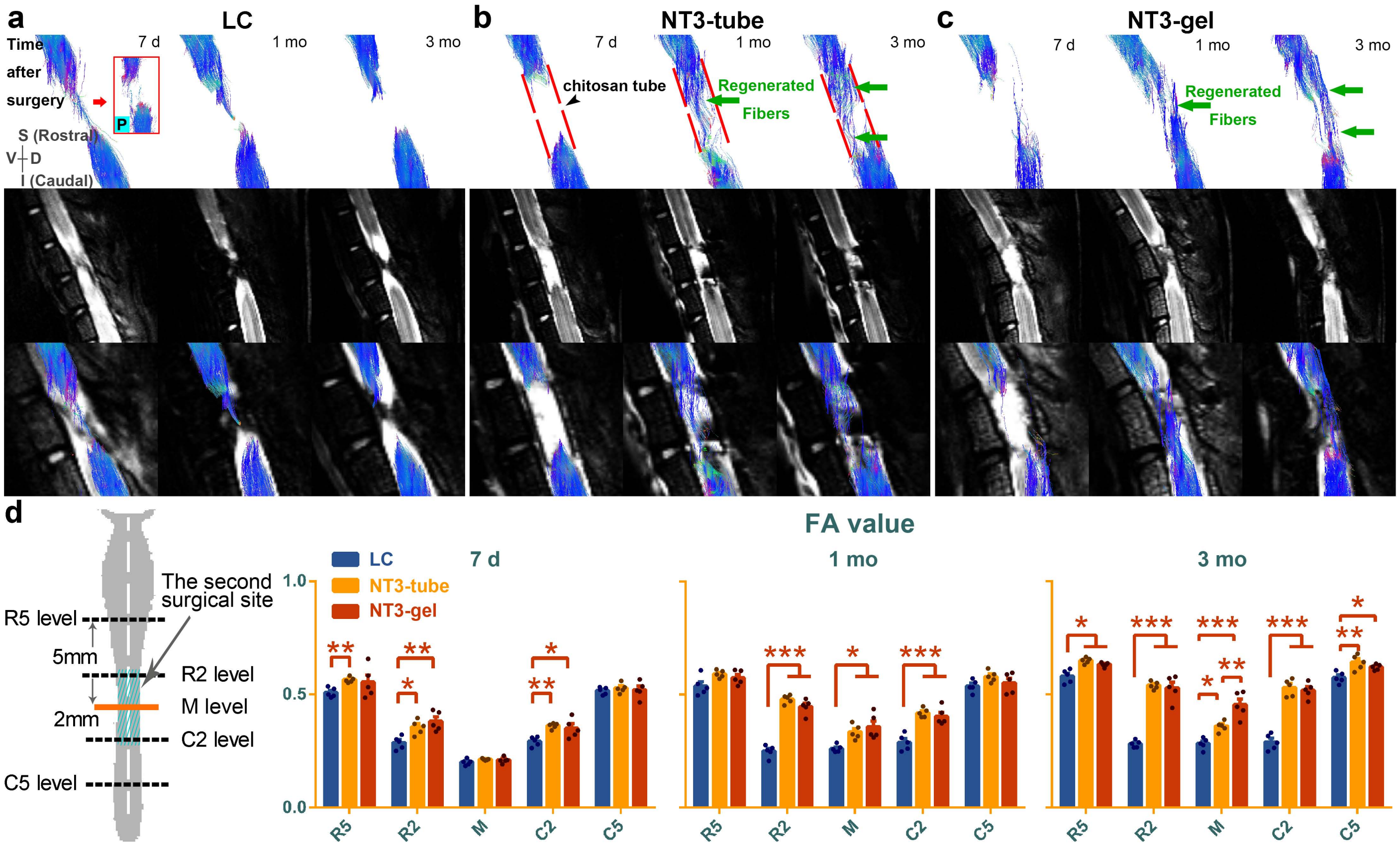

a
LC
Time after surgery
7 d
1 mo
3 mo
P
S (Rostral)
V D
I (Caudal)
b
NT3-tube
7 d
1 mo
3 mo
chitosan tube
Regenerated Fibers
c
NT3-gel
7 d
1 mo
3 mo
Regenerated Fibers
d
The second surgical site
R5 level
5mm
R2 level
2mm
M level
C2 level
C5 level
FA value
LC
NT3-tube
NT3-gel
7 d
1 mo
3 mo
1.0
0.5
0.0
R5
R2
M
C2
C5

Fig. S3. Diffusion tensor fiber tracking tractography showed longitudinal changes in spinal cord of three groups over time after the second surgery and comparisons of changes in FA values and spinal cord atrophy among three groups after the second surgery. LC (a) animals showed a lack of neural fibers in the damaged zone, while NT3-tube (b) and NT3-gel (c) animals displayed signals representing regenerated fiber bundles growth into the surgical site and reconnecting the two ends of severed cord. The middle panels are sagittal structural MRI images. The bottom panels are fiber tracks overlaid on the structural MRI images to show the regenerated fiber bundles passing through the damaged area. S, superior (rostral); I, inferior (caudal); V, ventral; D, dorsal. Fibers in red boxes demonstrated fiber tracking results observed from the dorsal-ventral view. Red dashed lines in the NT3-tube group represent positions of chitosan tubes. (d) Comparisons of changes in FA values of spinal cords at different positions from different groups were measured at 7 days, 1 month and 3 months after the second surgery. Diagram illustrates locations of sampling points. *, $P < 0.05$; **, $P < 0.01$; ***, $P < 0.001$, by ANOVA. Data are shown as mean ± SEM, $n = 5$. See Supplementary Table 2 for exact *P*-values and statistical tests.

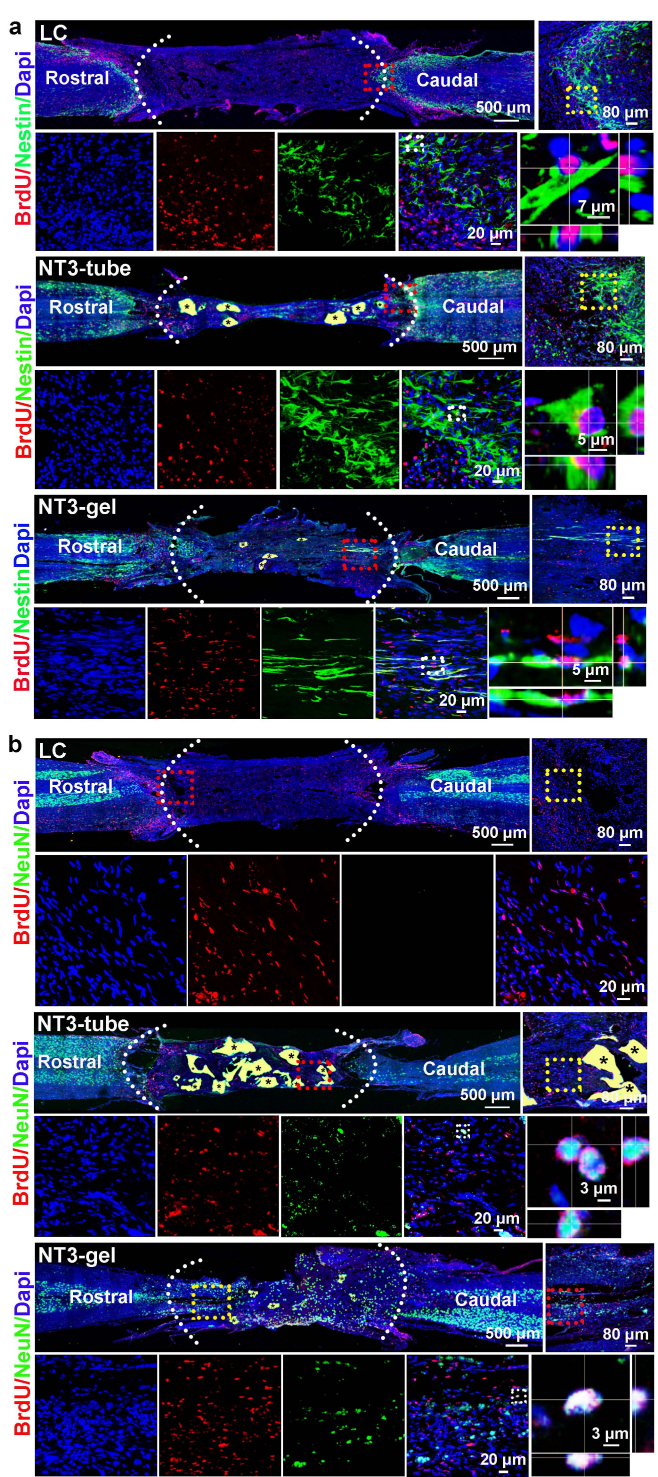
a
LC
Rostral
Caudal
500 µm
80 µm
BrdU/Nestin/Dapi
20 µm
7 µm
NT3-tube
Rostral
Caudal
500 µm
80 µm
BrdU/Nestin/Dapi
20 µm
5 µm
NT3-gel
Rostral
Caudal
500 µm
80 µm
BrdU/Nestin/Dapi
20 µm
5 µm
b
LC
Rostral
Caudal
500 µm
80 µm
BrdU/NeuN/Dapi
20 µm
NT3-tube
Rostral
Caudal
500 µm
BrdU/NeuN/Dapi
20 µm
3 µm
NT3-gel
Rostral
Caudal
500 µm
80 µm
BrdU/NeuN/Dapi
20 µm
3 µm

Fig. S4. NSCs and neurons were found in regenerating neural tissues. (a) Immunofluorescent staining of BrdU and nestin at three months after second operation, $BrdU^+/nestin^+$ cells were found in regenerating neural tissues 3 mo after the second operation. (b) $BrdU^+/NeuN^+$ cells were found in the regenerating neural tissues from NT3-tube and NT3-gel groups. No $BrdU^+/NeuN^+$ cells were detected in the LC group at 3 mo after the second operation. *Labels undegraded chitosan biomaterials.

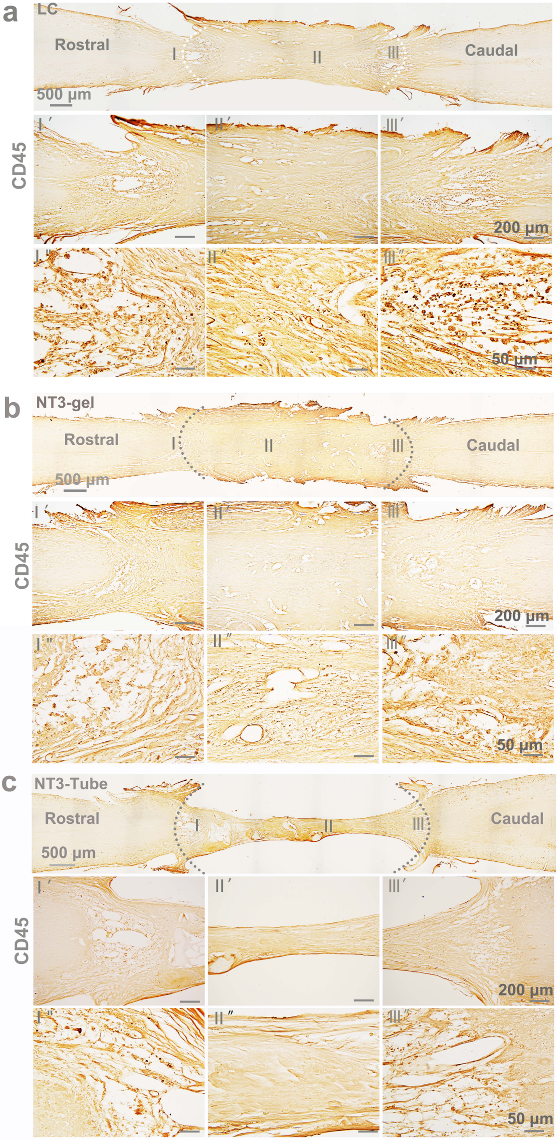
a
LC
Rostral
I
II
III
Caudal
500 μm
CD45
I′
II′
III′
200 μm
I″
II″
III″
50 μm
b
NT3-gel
Rostral
I
II
III
Caudal
500 μm
CD45
I′
II′
III′
200 μm
I″
II″
III″
50 μm
c
NT3-Tube
Rostral
I
II
III
Caudal
500 μm
CD45
I′
II′
III′
200 μm
I″
II″
III″
50 μm

Fig. S5. NT3-chitosan treatment reduced inflammation (CD45 labeling). (a) Immunohistochemical staining of CD45 in the LC group three months after the second operation. A large number of CD45 positive inflammatory cells were observed in the injury area. (b) and (c) reduced amount of CD45 positive inflammatory cells could be detected in NT3-tube and NT3-gel groups.

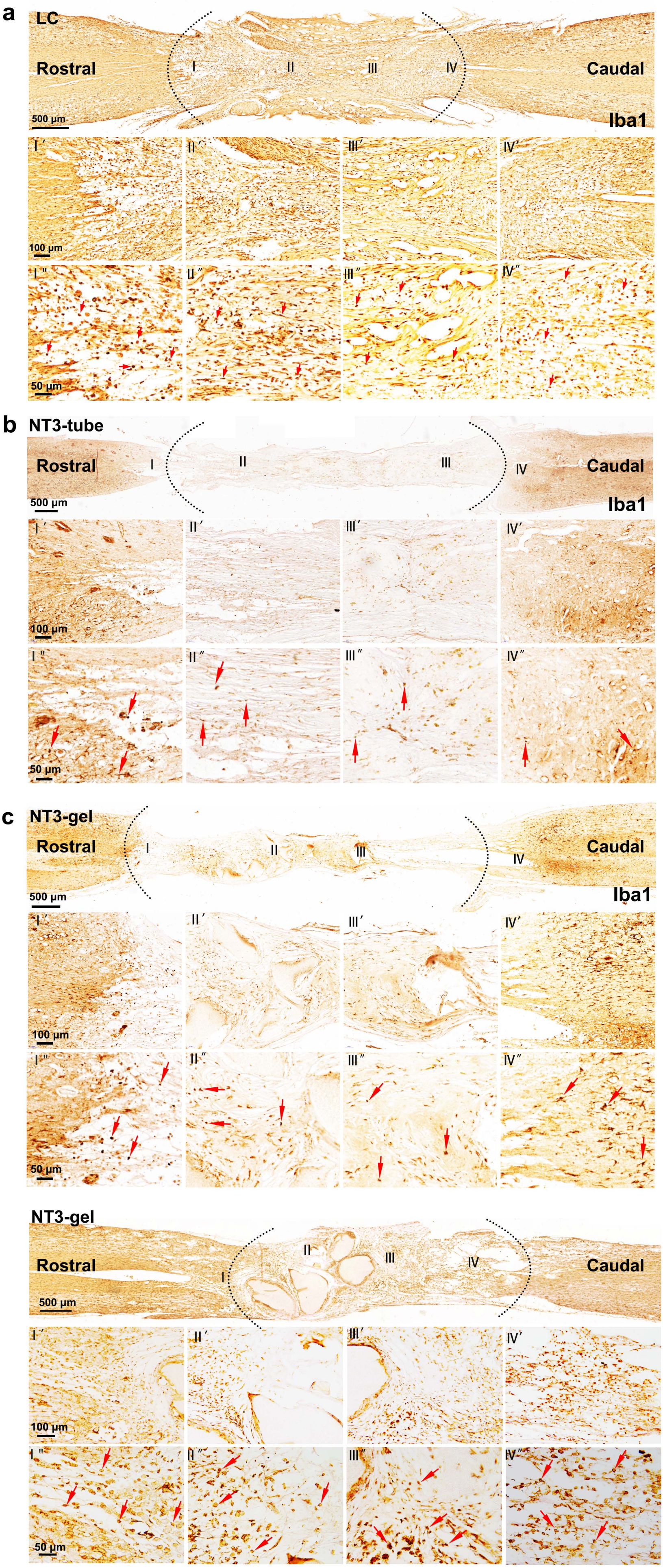

a
LC
Rostral
I
II
III
IV
Caudal
500 μm
Iba1
I′
II′
III′
IV′
100 μm
I″
II″
III″
IV″
50 μm
b
NT3-tube
Rostral
I
II
III
IV
Caudal
500 μm
Iba1
I′
II′
III′
IV′
100 μm
I″
II″
III″
IV″
50 μm
c
NT3-gel
Rostral
I
II
III
IV
Caudal
500 μm
Iba1
I′
II′
III′
IV′
100 μm
I″
II″
III″
IV″
50 μm
NT3-gel
Rostral
I
II
III
IV
Caudal
500 μm
I′
II′
III′
IV′
100 μm
I″
II″
III″
IV″
50 μm

Fig. S6. NT3-chitosan treatment reduced inflammation (IBA1 labeling). (a) Immunohistochemical staining of IBA1 in the LC group three months after the second operation. A large number of IBA1 positive inflammatory cells were observed in the injury area. (b) and (c) reduced amount of IBA1 positive inflammatory cells could be detected in NT3-tube and NT3-gel groups.

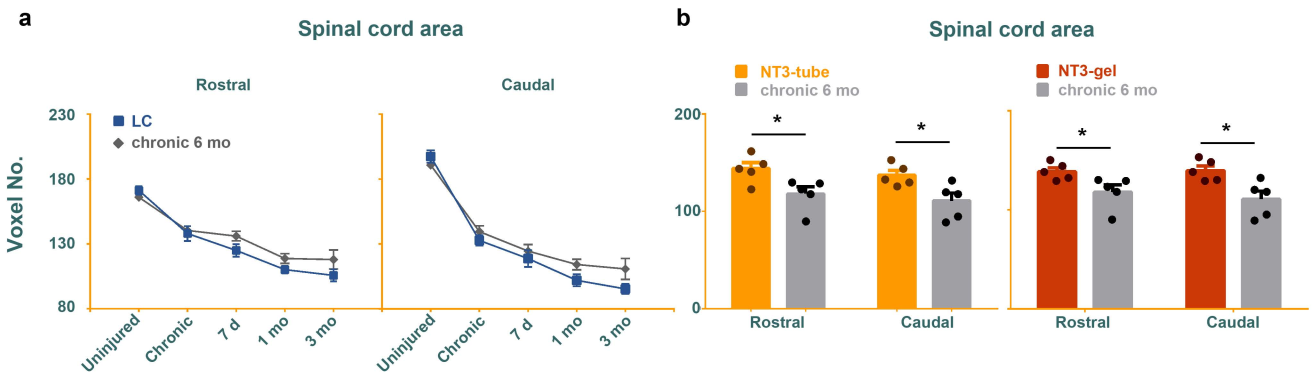

a
Spinal cord area
Rostral
Caudal
LC
chronic 6 mo
Voxel No.
230
180
130
80
Uninjured
Chronic
7 d
1 mo
3 mo
b
Spinal cord area
NT3-tube
chronic 6 mo
NT3-gel
chronic 6 mo
200
100
0
*
Rostral
Caudal

Fig. S7. Comparisons of changes in spinal cord atrophy between LC (3+3mo) and chronic 6 mo (without 2nd surgery) groups at 7day, 1 month, and 3 months after secondary surgery and equivalent times for the chronic group (a), and between NT3-tube/NT3-gel and chronic 6 mo groups at 6 months after the first surgery (b). *, $P < 0.05$, by two-tailed independent sample t test. Data are shown as mean ± SEM, $n = 5$. See Supplementary Table 2 for exact *P*-values and statistical tests.

SI Tables

Table.1 Matching degree of lesion area by MR-DTI and pathology.

Timepoints	Area			Width			Length		
	DTT (mm^2)	NF/GFAP (mm^2)	Matching rate (%)	DTT (mm)	NF/GFAP (mm)	Matching rate (%)	DTT (mm)	NF/GFAP (mm)	Matching rate (%)
10 d	1.764	2.035	86.671	1.764	1.751	99.232	1.0	1.345	74.364
20 d	2.793	2.918	95.707	1.470	1.910	76.953	2.0	2.118	94.434
1 mo	2.425	2.457	98.695	1.617	1.636	98.881	1.5	1.659	90.425
2 mo	2.940	2.977	98.750	1.323	1.405	94.193	2.0	2.176	91.900
3 mo	3.087	3.000	97.109	1.176	1.081	91.222	3.0	3.069	97.751

Note: The calculation method of the “matching rate” is provided in the “Materials and Methods”. d, days; mo, months.

Table S2. Exacted *P* values and statistical tests for each significant difference.

Table S2. Exacted P values and statistical tests for each significant difference.

Figure	p-value (comparison, test, estimate of variation)	degrees of freedom
Fig.3c	<0.0001 (Uninjured vs Chronic, ANOVA with Dunnetts' T3, variance inhomogeneous)	(44,4,40)
	<0.0001 (Uninjured vs LC, ANOVA with Dunnetts' T3, variance inhomogeneous)	
	<0.0001 (Uninjured vs NT3-tube, ANOVA with Dunnetts' T3, variance inhomogeneous)	
	0.003229 (Uninjured vs NT3-gel, ANOVA with Dunnetts' T3, variance inhomogeneous)	
	1.000000 (Chronic vs LC, ANOVA with Dunnetts' T3, variance inhomogeneous)	
	0.003563 (Chronic vs NT3-tube, ANOVA with Dunnetts' T3, variance inhomogeneous)	
	0.007144 (Chronic vs NT3-gel, ANOVA with Dunnetts' T3, variance inhomogeneous)	
	0.008456 (LC vs NT3-tube, ANOVA with Dunnetts' T3, variance inhomogeneous)	
	0.007936 (LC vs NT3-gel, ANOVA with Dunnetts' T3, variance inhomogeneous)	
	0.102711 (NT3-tube vs NT3-gel, ANOVA with Dunnetts' T3, variance inhomogeneous)	
Fig. 4d	Tuj1	
	0.707520 (R: NT3-tube vs NT3-gel, two-tailed Independent Sample T-Test, variance homogeneous)	8
	0.444132 (M: NT3-tube vs NT3-gel, two-tailed Independent Sample T-Test, variance homogeneous)	8
	0.616791 (C: NT3-tube vs NT3-gel, two-tailed Independent Sample T-Test, variance homogeneous)	8
	Nestin	
	0.000005 (R: LC vs NT3-tube, ANOVA with Dunnetts' T3, variance inhomogeneous)	(14,2,12)
	0.000009 (R: LC vs NT3-gel, ANOVA with Dunnetts' T3, variance inhomogeneous)	
	0.910939 (R: NT3-tube vs NT3-gel, ANOVA with Dunnetts' T3, variance inhomogeneous)	
	<0.0001 (M: LC vs NT3-tube, ANOVA with Bonferroni, variance homogeneous)	(14,2,12)
	<0.0001 (M: LC vs NT3-gel, ANOVA with Bonferroni, variance homogeneous)	
	1.000000 (M: NT3-tube vs NT3-gel, ANOVA with Bonferroni, variance homogeneous)	
	0.000010 (C: LC vs NT3-tube, ANOVA with Dunnetts' T3, variance inhomogeneous)	(14,2,12)
	0.000009 (C: LC vs NT3-gel, ANOVA with Dunnetts' T3, variance inhomogeneous)	
	0.999049 (C: NT3-tube vs NT3-gel, ANOVA with Dunnetts' T3, variance inhomogeneous)	
	NeuN	
	0.281359 (R: NT3-tube vs NT3-gel, two-tailed Independent Sample T-Test, variance homogeneous)	8
	0.153205 (M: NT3-tube vs NT3-gel, two-tailed Independent Sample T-Test, variance homogeneous)	8
	0.234402 (C: NT3-tube vs NT3-gel, two-tailed Independent Sample T-Test, variance homogeneous)	8
Fig.5b	spinal cord area:	
	1.000000 (Rostral, Uninjured: LC vs NT3-tube, ANOVA with Bonferroni, variance homogeneous)	(14,2,12)
	1.000000 (Rostral, Uninjured: LC vs NT3-gel, ANOVA with Bonferroni, variance homogeneous)	
	1.000000 (Rostral, Uninjured: NT3-tube vs NT3-gel, ANOVA with Bonferroni, variance homogeneous)	

1.000000 (Rostral, Chronic: LC vs NT3-tube, ANOVA with Bonferroni, variance homogeneous) (14,2,12)
1.000000 (Rostral, Chronic: LC vs NT3-gel, ANOVA with Bonferroni, variance homogeneous)
1.000000 (Rostral, Chronic: NT3-tube vs NT3-gel, ANOVA with Bonferroni, variance homogeneous)
0.423028 (Rostral, 7 d: LC vs NT3-tube, ANOVA with Bonferroni, variance homogeneous) (14,2,12)
1.000000 (Rostral, 7 d: LC vs NT3-gel, ANOVA with Bonferroni, variance homogeneous)
1.000000 (Rostral, 7 d: NT3-tube vs NT3-gel, ANOVA with Bonferroni, variance homogeneous)
0.000370 (Rostral, 1 mo: LC vs NT3-tube, ANOVA with Bonferroni, variance homogeneous) (14,2,12)
0.003269 (Rostral, 1 mo: LC vs NT3-gel, ANOVA with Bonferroni, variance homogeneous)
0.660356 (Rostral, 1 mo: NT3-tube vs NT3-gel, ANOVA with Bonferroni, variance homogeneous)
0.000520 (Rostral, 3 mo: LC vs NT3-tube, ANOVA with Bonferroni, variance homogeneous) (14,2,12)
0.001774 (Rostral, 3 mo: LC vs NT3-gel, ANOVA with Bonferroni, variance homogeneous)
1.000000 (Rostral, 3 mo: NT3-tube vs NT3-gel, ANOVA with Bonferroni, variance homogeneous)
0.147048 (Caudal, Uninjured: LC vs NT3-tube, ANOVA with Bonferroni, variance homogeneous) (14,2,12)
1.000000 (Caudal, Uninjured: LC vs NT3-gel, ANOVA with Bonferroni, variance homogeneous)
0.298309 (Caudal, Uninjured: NT3-tube vs NT3-gel, ANOVA with Bonferroni, variance homogeneous)
1.000000 (Caudal, Chronic: LC vs NT3-tube, ANOVA with Bonferroni, variance homogeneous) (14,2,12)
1.000000 (Caudal, Chronic: LC vs NT3-gel, ANOVA with Bonferroni, variance homogeneous)
1.000000 (Caudal, Chronic: NT3-tube vs NT3-gel, ANOVA with Bonferroni, variance homogeneous)
0.284687 (Caudal, 7 d: LC vs NT3-tube, ANOVA with Bonferroni, variance homogeneous) (14,2,12)
1.000000 (Caudal, 7 d: LC vs NT3-gel, ANOVA with Bonferroni, variance homogeneous)
1.000000 (Caudal, 7 d: NT3-tube vs NT3-gel, ANOVA with Bonferroni, variance homogeneous)
0.000308 (Caudal, 1 mo: LC vs NT3-tube, ANOVA with Bonferroni, variance homogeneous) (14,2,12)
0.000558 (Caudal, 1 mo: LC vs NT3-gel, ANOVA with Bonferroni, variance homogeneous)
1.000000 (Caudal, 1 mo: NT3-tube vs NT3-gel, ANOVA with Bonferroni, variance homogeneous)
0.000089 (Caudal, 3 mo: LC vs NT3-tube, ANOVA with Bonferroni, variance homogeneous) (14,2,12)
0.000052 (Caudal, 3 mo: LC vs NT3-gel, ANOVA with Bonferroni, variance homogeneous)
1.000000 (Caudal, 3 mo: NT3-tube vs NT3-gel, ANOVA with Bonferroni, variance homogeneous)
Dorsal-ventral axis
1.000000 (Rostral, Uninjured: LC vs NT3-tube, ANOVA with Bonferroni, variance homogeneous) (14,2,12)
1.000000 (Rostral, Uninjured: LC vs NT3-gel, ANOVA with Bonferroni, variance homogeneous)
1.000000 (Rostral, Uninjured: NT3-tube vs NT3-gel, ANOVA with Bonferroni, variance homogeneous)
1.000000 (Rostral, Chronic: LC vs NT3-tube, ANOVA with Bonferroni, variance homogeneous) (14,2,12)
0.732510 (Rostral, Chronic: LC vs NT3-gel, ANOVA with Bonferroni, variance homogeneous)
1.000000 (Rostral, Chronic: NT3-tube vs NT3-gel, ANOVA with Bonferroni, variance homogeneous)
0.378822 (Rostral, 7 d: LC vs NT3-tube, ANOVA with Bonferroni, variance homogeneous) (14,2,12)
1.000000 (Rostral, 7 d: LC vs NT3-gel, ANOVA with Bonferroni, variance homogeneous)

0.884462 (Rostral, 7 d: NT3-tube vs NT3-gel, ANOVA with Bonferroni, variance homogeneous)
0.000154 (Rostral, 1 mo: LC vs NT3-tube, ANOVA with Bonferroni, variance homogeneous) (14,2,12)
0.003170 (Rostral, 1 mo: LC vs NT3-gel, ANOVA with Bonferroni, variance homogeneous)
0.273212 (Rostral, 1 mo: NT3-tube vs NT3-gel, ANOVA with Bonferroni, variance homogeneous)
0.000129 (Rostral, 3 mo: LC vs NT3-tube, ANOVA with Bonferroni, variance homogeneous) (14,2,12)
0.001292 (Rostral, 3 mo: LC vs NT3-gel, ANOVA with Bonferroni, variance homogeneous)
0.525369 (Rostral, 3 mo: NT3-tube vs NT3-gel, ANOVA with Bonferroni, variance homogeneous)
0.153736 (Caudal, Uninjured: LC vs NT3-tube, ANOVA with Bonferroni, variance homogeneous) (14,2,12)
1.000000 (Caudal, Uninjured: LC vs NT3-gel, ANOVA with Bonferroni, variance homogeneous)
0.655010 (Caudal, Uninjured: NT3-tube vs NT3-gel, ANOVA with Bonferroni, variance homogeneous)
1.000000 (Caudal, Chronic: LC vs NT3-tube, ANOVA with Bonferroni, variance homogeneous) (14,2,12)
1.000000 (Caudal, Chronic: LC vs NT3-gel, ANOVA with Bonferroni, variance homogeneous)
1.000000 (Caudal, Chronic: NT3-tube vs NT3-gel, ANOVA with Bonferroni, variance homogeneous)
0.803601 (Caudal, 7 d: LC vs NT3-tube, ANOVA with Bonferroni, variance homogeneous) (14,2,12)
1.000000 (Caudal, 7 d: LC vs NT3-gel, ANOVA with Bonferroni, variance homogeneous)
1.000000 (Caudal, 7 d: NT3-tube vs NT3-gel, ANOVA with Bonferroni, variance homogeneous)
0.015902 (Caudal, 1 mo: LC vs NT3-tube, ANOVA with Bonferroni, variance homogeneous) (14,2,12)
0.004609 (Caudal, 1 mo: LC vs NT3-gel, ANOVA with Bonferroni, variance homogeneous)
1.000000 (Caudal, 1 mo: NT3-tube vs NT3-gel, ANOVA with Bonferroni, variance homogeneous)
0.000269 (Caudal, 3 mo: LC vs NT3-tube, ANOVA with Bonferroni, variance homogeneous) (14,2,12)
0.000017 (Caudal, 3 mo: LC vs NT3-gel, ANOVA with Bonferroni, variance homogeneous)
0.236190 (Caudal, 3 mo: NT3-tube vs NT3-gel, ANOVA with Bonferroni, variance homogeneous)
Left-right axis
0.296671 (Rostral, Uninjured: LC vs NT3-tube, ANOVA with Bonferroni, variance homogeneous) (14,2,12)
1.000000 (Rostral, Uninjured: LC vs NT3-gel, ANOVA with Bonferroni, variance homogeneous)
0.613650 (Rostral, Uninjured: NT3-tube vs NT3-gel, ANOVA with Bonferroni, variance homogeneous)
1.000000 (Rostral, Chronic: LC vs NT3-tube, ANOVA with Bonferroni, variance homogeneous) (14,2,12)
1.000000 (Rostral, Chronic: LC vs NT3-gel, ANOVA with Bonferroni, variance homogeneous)
1.000000 (Rostral, Chronic: NT3-tube vs NT3-gel, ANOVA with Bonferroni, variance homogeneous)
0.080653 (Rostral, 7 d: LC vs NT3-tube, ANOVA with Bonferroni, variance homogeneous) (14,2,12)
1.000000 (Rostral, 7 d: LC vs NT3-gel, ANOVA with Bonferroni, variance homogeneous)
0.080653 (Rostral, 7 d: NT3-tube vs NT3-gel, ANOVA with Bonferroni, variance homogeneous)
0.003321 (Rostral, 1 mo: LC vs NT3-tube, ANOVA with Bonferroni, variance homogeneous) (14,2,12)
0.027653 (Rostral, 1 mo: LC vs NT3-gel, ANOVA with Bonferroni, variance homogeneous)
0.803601 (Rostral, 1 mo: NT3-tube vs NT3-gel, ANOVA with Bonferroni, variance homogeneous)
0.022214 (Rostral, 3 mo: LC vs NT3-tube, ANOVA with Bonferroni, variance homogeneous) (14,2,12)

	0.012258 (Rostral, 3 mo: LC vs NT3-gel, ANOVA with Bonferroni, variance homogeneous)	
	1.000000 (Rostral, 3 mo: NT3-tube vs NT3-gel, ANOVA with Bonferroni, variance homogeneous)	
	1.000000 (Caudal, Uninjured: LC vs NT3-tube, ANOVA with Bonferroni, variance homogeneous)	(14,2,12)
	1.000000 (Caudal, Uninjured: LC vs NT3-gel, ANOVA with Bonferroni, variance homogeneous)	
	0.934583 (Caudal, Uninjured: NT3-tube vs NT3-gel, ANOVA with Bonferroni, variance homogeneous)	
	1.000000 (Caudal, Chronic: LC vs NT3-tube, ANOVA with Bonferroni, variance homogeneous)	(14,2,12)
	1.000000 (Caudal, Chronic: LC vs NT3-gel, ANOVA with Bonferroni, variance homogeneous)	
	1.000000 (Caudal, Chronic: NT3-tube vs NT3-gel, ANOVA with Bonferroni, variance homogeneous)	
	0.603453 (Caudal, 7 d: LC vs NT3-tube, ANOVA with Bonferroni, variance homogeneous)	(14,2,12)
	1.000000 (Caudal, 7 d: LC vs NT3-gel, ANOVA with Bonferroni, variance homogeneous)	
	1.000000 (Caudal, 7 d: NT3-tube vs NT3-gel, ANOVA with Bonferroni, variance homogeneous)	
	0.000312 (Caudal, 1 mo: LC vs NT3-tube, ANOVA with Bonferroni, variance homogeneous)	(14,2,12)
	0.001073 (Caudal, 1 mo: LC vs NT3-gel, ANOVA with Bonferroni, variance homogeneous)	
	1.000000 (Caudal, 1 mo: NT3-tube vs NT3-gel, ANOVA with Bonferroni, variance homogeneous)	
	0.005957 (Caudal, 3 mo: LC vs NT3-tube, ANOVA with Bonferroni, variance homogeneous)	(14,2,12)
	0.003601 (Caudal, 3 mo: LC vs NT3-gel, ANOVA with Bonferroni, variance homogeneous)	
	0.236190 (Caudal, 3 mo: NT3-tube vs NT3-gel, ANOVA with Bonferroni, variance homogeneous)	
Fig.6a	0.060296 (Latency, L: Uninjured vs NT3-tube, ANOVA with Bonferroni, variance homogeneous)	(11,2,9)
	0.063757 (Latency, L: Uninjured vs NT3-gel, ANOVA with Bonferroni, variance homogeneous)	
	1.000000 (Latency, L: NT3-tube vs NT3-gel, ANOVA with Bonferroni, variance homogeneous)	
	0.064096 (Latency, R: Uninjured vs NT3-tube, ANOVA with Bonferroni, variance homogeneous)	(11,2,9)
	0.160293 (Latency, R: Uninjured vs NT3-gel, ANOVA with Bonferroni, variance homogeneous)	
	1.000000 (Latency, R: NT3-tube vs NT3-gel, ANOVA with Bonferroni, variance homogeneous)	
	0.004701 (Amplitude, L: Uninjured vs NT3-tube, ANOVA with Bonferroni, variance homogeneous)	(11,2,9)
	0.002684 (Amplitude, L: Uninjured vs NT3-gel, ANOVA with Bonferroni, variance homogeneous)	
	1.000000 (Amplitude, L: NT3-tube vs NT3-gel, ANOVA with Bonferroni, variance homogeneous)	
	0.005508 (Amplitude, R: Uninjured vs NT3-tube, ANOVA with Bonferroni, variance homogeneous)	(11,2,9)
	0.003115 (Amplitude, R: Uninjured vs NT3-gel, ANOVA with Bonferroni, variance homogeneous)	
	1.000000 (Amplitude, R: NT3-tube vs NT3-gel, ANOVA with Bonferroni, variance homogeneous)	
Fig.6b	0.477000 (Latency, L: Uninjured vs NT3-tube, ANOVA with Bonferroni, variance homogeneous)	(11,2,9)
	0.582801 (Latency, L: Uninjured vs NT3-gel, ANOVA with Bonferroni, variance homogeneous)	
	1.000000 (Latency, L: NT3-tube vs NT3-gel, ANOVA with Bonferroni, variance homogeneous)	
	0.083300 (Latency, R: Uninjured vs NT3-tube, ANOVA with Bonferroni, variance homogeneous)	(11,2,9)
	0.247468 (Latency, R: Uninjured vs NT3-gel, ANOVA with Bonferroni, variance homogeneous)	

	1.000000 (Latency, R: NT3-tube vs NT3-gel, ANOVA with Bonferroni, variance homogeneous)	
	0.000120 (Amplitude, L: Uninjured vs NT3-tube, ANOVA with Bonferroni, variance homogeneous)	(11,2,9)
	0.000061 (Amplitude, L: Uninjured vs NT3-gel, ANOVA with Bonferroni, variance homogeneous)	
	1.000000 (Amplitude, L: NT3-tube vs NT3-gel, ANOVA with Bonferroni, variance homogeneous)	
	0.000106 (Amplitude, R: Uninjured vs NT3-tube, ANOVA with Bonferroni, variance homogeneous)	(11,2,9)
	0.000067 (Amplitude, R: Uninjured vs NT3-gel, ANOVA with Bonferroni, variance homogeneous)	
	1.000000 (Amplitude, R: NT3-tube vs NT3-gel, ANOVA with Bonferroni, variance homogeneous)	
Fig.6c	1.000000 (first surgery 1w: LC vs NT3-tube, ANOVA with Bonferroni, variance homogeneous)	(23,2,21)
	1.000000 (first surgery 1w: LC vs NT3-gel, ANOVA with Bonferroni, variance homogeneous)	
	1.000000 (first surgery 1w: NT3-tube vs NT3-gel, ANOVA with Bonferroni, variance homogeneous)	
	1.000000 (first surgery 2w: LC vs NT3-tube, ANOVA with Bonferroni, variance homogeneous)	(23,2,21)
	1.000000 (first surgery 2w: LC vs NT3-gel, ANOVA with Bonferroni, variance homogeneous)	
	1.000000 (first surgery 2w: NT3-tube vs NT3-gel, ANOVA with Bonferroni, variance homogeneous)	
	1.000000 (first surgery 4w: LC vs NT3-tube, ANOVA with Bonferroni, variance homogeneous)	(23,2,21)
	1.000000 (first surgery 4w: LC vs NT3-gel, ANOVA with Bonferroni, variance homogeneous)	
	1.000000 (first surgery 4w: NT3-tube vs NT3-gel, ANOVA with Bonferroni, variance homogeneous)	
	1.000000 (first surgery 8w: LC vs NT3-tube, ANOVA with Bonferroni, variance homogeneous)	(23,2,21)
	1.000000 (first surgery 8w: LC vs NT3-gel, ANOVA with Bonferroni, variance homogeneous)	
	1.000000 (first surgery 8w: NT3-tube vs NT3-gel, ANOVA with Bonferroni, variance homogeneous)	
	1.000000 (first surgery 12w: LC vs NT3-tube, ANOVA with Bonferroni, variance homogeneous)	(23,2,21)
	1.000000 (first surgery 12w: LC vs NT3-gel, ANOVA with Bonferroni, variance homogeneous)	
	1.000000 (first surgery 12w: NT3-tube vs NT3-gel, ANOVA with Bonferroni, variance homogeneous)	
	1.000000 (second surgery 1w: LC vs NT3-tube, ANOVA with Bonferroni, variance homogeneous)	(23,2,21)
	1.000000 (second surgery 1w: LC vs NT3-gel, ANOVA with Bonferroni, variance homogeneous)	
	1.000000 (second surgery 1w: NT3-tube vs NT3-gel, ANOVA with Bonferroni, variance homogeneous)	
	0.003226 (second surgery 2w: LC vs NT3-tube, ANOVA with Bonferroni, variance homogeneous)	(23,2,21)
	0.003226 (second surgery 2w: LC vs NT3-gel, ANOVA with Bonferroni, variance homogeneous)	
	1.000000 (second surgery 2w: NT3-tube vs NT3-gel, ANOVA with Bonferroni, variance homogeneous)	
	0.004214 (second surgery 3w: LC vs NT3-tube, ANOVA with Bonferroni, variance homogeneous)	(23,2,21)
	0.001900 (second surgery 3w: LC vs NT3-gel, ANOVA with Bonferroni, variance homogeneous)	
	1.000000 (second surgery 3w: NT3-tube vs NT3-gel, ANOVA with Bonferroni, variance homogeneous)	
	0.000038 (second surgery 4w: LC vs NT3-tube, ANOVA with Bonferroni, variance homogeneous)	(23,2,21)
	0.000105 (second surgery 4w: LC vs NT3-gel, ANOVA with Bonferroni, variance homogeneous)	
	1.000000 (second surgery 4w: NT3-tube vs NT3-gel, ANOVA with Bonferroni, variance homogeneous)	
	<0.0001 (second surgery 6w: LC vs NT3-tube, ANOVA with Bonferroni, variance homogeneous)	(23,2,21)

<0.0001 (second surgery 6w: LC vs NT3-gel, ANOVA with Bonferroni, variance homogeneous)
1.000000 (second surgery 6w: NT3-tube vs NT3-gel, ANOVA with Bonferroni, variance homogeneous)
<0.0001 (second surgery 8w: LC vs NT3-tube, ANOVA with Bonferroni, variance homogeneous) (23,2,21)
<0.0001 (second surgery 8w: LC vs NT3-gel, ANOVA with Bonferroni, variance homogeneous)
1.000000 (second surgery 8w: NT3-tube vs NT3-gel, ANOVA with Bonferroni, variance homogeneous)
<0.0001 (second surgery 10w: LC vs NT3-tube, ANOVA with Bonferroni, variance homogeneous) (23,2,21)
<0.0001 (second surgery 10w: LC vs NT3-gel, ANOVA with Bonferroni, variance homogeneous)
1.000000 (second surgery 10w: NT3-tube vs NT3-gel, ANOVA with Bonferroni, variance homogeneous)
<0.0001 (second surgery 12w: LC vs NT3-tube, ANOVA with Dunnetts' T3, variance inhomogeneous) (23,2,21)
<0.0001 (second surgery 12w: LC vs NT3-gel, ANOVA with Dunnetts' T3, variance inhomogeneous)
0.888275 (second surgery 12w: NT3-tube vs NT3-gel, ANOVA with Dunnetts' T3, variance inhomogeneous)
1.000000 (recut new 1w: LC vs NT3-tube, ANOVA with Bonferroni, variance homogeneous) (23,2,21)
1.000000 (recut new 1w: LC vs NT3-gel, ANOVA with Bonferroni, variance homogeneous)
1.000000 (recut new 1w: NT3-tube vs NT3-gel, ANOVA with Bonferroni, variance homogeneous)
1.000000 (recut new 2w: LC vs NT3-tube, ANOVA with Bonferroni, variance homogeneous) (23,2,21)
1.000000 (recut new 2w: LC vs NT3-gel, ANOVA with Bonferroni, variance homogeneous)
1.000000 (recut new 2w: NT3-tube vs NT3-gel, ANOVA with Bonferroni, variance homogeneous)
1.000000 (recut new 3w: LC vs NT3-tube, ANOVA with Bonferroni, variance homogeneous) (23,2,21)
1.000000 (recut new 3w: LC vs NT3-gel, ANOVA with Bonferroni, variance homogeneous)
1.000000 (recut new 3w: NT3-tube vs NT3-gel, ANOVA with Bonferroni, variance homogeneous)
1.000000 (recut new 4w: LC vs NT3-tube, ANOVA with Bonferroni, variance homogeneous) (23,2,21)
1.000000 (recut new 4w: LC vs NT3-gel, ANOVA with Bonferroni, variance homogeneous)
1.000000 (recut new 4w: NT3-tube vs NT3-gel, ANOVA with Bonferroni, variance homogeneous)

Fig.6d 1.000000 (second surgery 1w: NT3-tube (Acute) vs NT3-tube, ANOVA with Bonferroni, variance homogeneous) (23,2,21)
1.000000 (second surgery 1w: NT3-tube (Acute) vs NT3-gel, ANOVA with Bonferroni, variance homogeneous)
1.000000 (second surgery 1w: NT3-tube vs NT3-gel, ANOVA with Bonferroni, variance homogeneous)
0.002760 (second surgery 2w: NT3-tube (Acute) vs NT3-tube, ANOVA with Bonferroni, variance homogeneous) (23,2,21)
0.002760 (second surgery 2w: NT3-tube (Acute) vs NT3-gel, ANOVA with Bonferroni, variance homogeneous)
1.000000 (second surgery 2w: NT3-tube vs NT3-gel, ANOVA with Bonferroni, variance homogeneous)
0.002270 (second surgery 3w: NT3-tube (Acute) vs NT3-tube, ANOVA with Bonferroni, variance homogeneous) (23,2,21)
0.005316 (second surgery 3w: NT3-tube (Acute) vs NT3-gel, ANOVA with Bonferroni, variance homogeneous)
1.000000 (second surgery 3w: NT3-tube vs NT3-gel, ANOVA with Bonferroni, variance homogeneous)
0.035290 (second surgery 4w: NT3-tube (Acute) vs NT3-tube, ANOVA with Bonferroni, variance homogeneous) (23,2,21)
0.013076 (second surgery 4w: NT3-tube (Acute) vs NT3-gel, ANOVA with Bonferroni, variance homogeneous)
1.000000 (second surgery 4w: NT3-tube vs NT3-gel, ANOVA with Bonferroni, variance homogeneous)

0.017294 (second surgery 6w: NT3-tube (Acute) vs NT3-tube, ANOVA with Bonferroni, variance homogeneous) (23,2,21)
0.006510 (second surgery 6w: NT3-tube (Acute) vs NT3-gel, ANOVA with Bonferroni, variance homogeneous)
1.000000 (second surgery 6w: NT3-tube vs NT3-gel, ANOVA with Bonferroni, variance homogeneous)
0.016777 (second surgery 8w: NT3-tube (Acute) vs NT3-tube, ANOVA with Bonferroni, variance homogeneous) (23,2,21)
0.041677 (second surgery 8w: NT3-tube (Acute) vs NT3-gel, ANOVA with Bonferroni, variance homogeneous)
1.000000 (second surgery 8w: NT3-tube vs NT3-gel, ANOVA with Bonferroni, variance homogeneous)
0.067436 (second surgery 10w: NT3-tube (Acute) vs NT3-tube, ANOVA with Bonferroni, variance homogeneous) (23,2,21)
0.052298 (second surgery 10w: NT3-tube (Acute) vs NT3-gel, ANOVA with Bonferroni, variance homogeneous)
1.000000 (second surgery 10w: NT3-tube vs NT3-gel, ANOVA with Bonferroni, variance homogeneous)
0.372098 (second surgery 12w: NT3-tube (Acute) vs NT3-tube, ANOVA with Bonferroni, variance homogeneous) (23,2,21)
0.103278 (second surgery 12w: NT3-tube (Acute) vs NT3-gel, ANOVA with Bonferroni, variance homogeneous)
1.000000 (second surgery 12w: NT3-tube vs NT3-gel, ANOVA with Bonferroni, variance homogeneous)

Fig.6f Nestin
<0.0001 (R: LC vs NT3-tube, ANOVA with Bonferroni, variance homogeneous) (19,3,16)
<0.0001 (R: LC vs NT3-gel, ANOVA with Bonferroni, variance homogeneous)
<0.0001 (R: LC vs NT3-tube (Acute), ANOVA with Bonferroni, variance homogeneous)
1.000000 (R: NT3-tube vs NT3-gel, ANOVA with Bonferroni, variance homogeneous)
0.003381 (R: NT3-tube vs NT3-tube (Acute), ANOVA with Bonferroni, variance homogeneous)
0.013538 (R: NT3-gel vs NT3-tube (Acute), ANOVA with Bonferroni, variance homogeneous
1.000000 (M: NT3-tube vs NT3-gel, ANOVA with Bonferroni, variance homogeneous) (14,2,12)
0.030256 (M: NT3-tube vs NT3-tube (Acute), ANOVA with Bonferroni, variance homogeneous)
0.005629 (M: NT3-gel vs NT3-tube (Acute), ANOVA with Bonferroni, variance homogeneous
<0.0001 (C: LC vs NT3-tube, ANOVA with Bonferroni, variance homogeneous) (19,3,16)
<0.0001 (C: LC vs NT3-gel, ANOVA with Bonferroni, variance homogeneous)
<0.0001 (C: LC vs NT3-tube (Acute), ANOVA with Bonferroni, variance homogeneous)
1.000000 (C: NT3-tube vs NT3-gel, ANOVA with Bonferroni, variance homogeneous)
0.000422 (C: NT3-tube vs NT3-tube (Acute), ANOVA with Bonferroni, variance homogeneous)
0.001327 (C: NT3-gel vs NT3-tube (Acute), ANOVA with Bonferroni, variance homogeneous
Tubulin
1.000000 (R: NT3-tube vs NT3-gel, ANOVA with Bonferroni, variance homogeneous) (14,2,12)
0.036947 (R: NT3-tube vs NT3-tube (Acute), ANOVA with Bonferroni, variance homogeneous)
0.016289 (R: NT3-gel vs NT3-tube (Acute), ANOVA with Bonferroni, variance homogeneous
1.000000 (M: NT3-tube vs NT3-gel, ANOVA with Bonferroni, variance homogeneous) (14,2,12)
0.001770 (M: NT3-tube vs NT3-tube (Acute), ANOVA with Bonferroni, variance homogeneous)
0.007272 (M: NT3-gel vs NT3-tube (Acute), ANOVA with Bonferroni, variance homogeneous

1.000000 (C: NT3-tube vs NT3-gel, ANOVA with Bonferroni, variance homogeneous) (14,2,12)
0.010733 (C: NT3-tube vs NT3-tube (Acute), ANOVA with Bonferroni, variance homogeneous)
0.003283 (C: NT3-gel vs NT3-tube (Acute), ANOVA with Bonferroni, variance homogeneous
NeuN
1.000000 (R: NT3-tube vs NT3-gel, ANOVA with Bonferroni, variance homogeneous) (14,2,12)
0.004065 (R: NT3-tube vs NT3-tube (Acute), ANOVA with Bonferroni, variance homogeneous)
0.007066 (R: NT3-gel vs NT3-tube (Acute), ANOVA with Bonferroni, variance homogeneous
1.000000 (M: NT3-tube vs NT3-gel, ANOVA with Bonferroni, variance homogeneous) (14,2,12)
0.008812 (M: NT3-tube vs NT3-tube (Acute), ANOVA with Bonferroni, variance homogeneous)
0.008812 (M: NT3-gel vs NT3-tube (Acute), ANOVA with Bonferroni, variance homogeneous
1.000000 (C: NT3-tube vs NT3-gel, ANOVA with Bonferroni, variance homogeneous) (14,2,12)
0.026584 (C: NT3-tube vs NT3-tube (Acute), ANOVA with Bonferroni, variance homogeneous)
0.045023 (C: NT3-gel vs NT3-tube (Acute), ANOVA with Bonferroni, variance homogeneous

Fig.S3d 0.004631 (7 d, R5: LC vs NT3-tube, ANOVA with Dunnetts' T3, variance inhomogeneous) (14,2,12)
0.481150 (7 d, R5: LC vs NT3-gel, ANOVA with Dunnetts' T3, variance inhomogeneous)
0.975615 (7 d, R5: NT3-tube vs NT3-gel, ANOVA with Dunnetts' T3, variance inhomogeneous)
0.029077 (7 d, R2: LC vs NT3-tube, ANOVA with Bonferroni, variance homogeneous) (14,2,12)
0.003597 (7 d, R2: LC vs NT3-gel, ANOVA with Bonferroni, variance homogeneous)
0.824936 (7 d, R2: NT3-tube vs NT3-gel, ANOVA with Bonferroni, variance homogeneous)
0.296921 (7 d, M: LC vs NT3-tube, ANOVA with Bonferroni, variance homogeneous) (14,2,12)
0.676868 (7 d, M: LC vs NT3-gel, ANOVA with Bonferroni, variance homogeneous)
1.000000 (7 d, M: NT3-tube vs NT3-gel, ANOVA with Bonferroni, variance homogeneous)
0.006472 (7 d, C2: LC vs NT3-tube, ANOVA with Bonferroni, variance homogeneous) (14,2,12)
0.022573 (7 d, C2: LC vs NT3-gel, ANOVA with Bonferroni, variance homogeneous)
1.000000 (7 d, C2: NT3-tube vs NT3-gel, ANOVA with Bonferroni, variance homogeneous)
1.000000 (7 d, C5: LC vs NT3-tube, ANOVA with Bonferroni, variance homogeneous) (14,2,12)
1.000000 (7 d, C5: LC vs NT3-gel, ANOVA with Bonferroni, variance homogeneous)
1.000000 (7 d, C5: NT3-tube vs NT3-gel, ANOVA with Bonferroni, variance homogeneous)

0.070221 (1 mo, R5: LC vs NT3-tube, ANOVA with Bonferroni, variance homogeneous) (14,2,12)
0.288041 (1 mo, R5: LC vs NT3-gel, ANOVA with Bonferroni, variance homogeneous)
1.000000 (1 mo, R5: NT3-tube vs NT3-gel, ANOVA with Bonferroni, variance homogeneous)
<0.0001 (1 mo, R2: LC vs NT3-tube, ANOVA with Bonferroni, variance homogeneous) (14,2,12)
<0.0001 (1 mo, R2: LC vs NT3-gel, ANOVA with Bonferroni, variance homogeneous)
0.211606 (1 mo, R2: NT3-tube vs NT3-gel, ANOVA with Bonferroni, variance homogeneous)

0.011297 (1 mo, M: LC vs NT3-tube, ANOVA with Dunnetts' T3, variance inhomogeneous) (14,2,12)
0.039852 (1 mo, M: LC vs NT3-gel, ANOVA with Dunnetts' T3, variance inhomogeneous)
0.806020 (1 mo, M: NT3-tube vs NT3-gel, ANOVA with Dunnetts' T3, variance inhomogeneous)
0.000111 (1 mo, C2: LC vs NT3-tube, ANOVA with Bonferroni, variance homogeneous) (14,2,12)
0.000334 (1 mo, C2: LC vs NT3-gel, ANOVA with Bonferroni, variance homogeneous)
1.000000 (1 mo, C2: NT3-tube vs NT3-gel, ANOVA with Bonferroni, variance homogeneous)
0.202087 (1 mo, C5: LC vs NT3-tube, ANOVA with Bonferroni, variance homogeneous) (14,2,12)
1.000000 (1 mo, C5: LC vs NT3-gel, ANOVA with Bonferroni, variance homogeneous)
0.645206 (1 mo, C5: NT3-tube vs NT3-gel, ANOVA with Bonferroni, variance homogeneous)

0.010875 (3 mo, R5: LC vs NT3-tube, ANOVA with Dunnetts' T3, variance inhomogeneous) (14,2,12)
0.041725 (3 mo, R5: LC vs NT3-gel, ANOVA with Dunnetts' T3, variance inhomogeneous)
0.111063 (3 mo, R5: NT3-tube vs NT3-gel, ANOVA with Dunnetts' T3, variance inhomogeneous)
<0.0001 (3 mo, R2: LC vs NT3-tube, ANOVA with Bonferroni, variance homogeneous) (14,2,12)
<0.0001 (3 mo, R2: LC vs NT3-gel, ANOVA with Bonferroni, variance homogeneous)
1.000000 (3 mo, R2: NT3-tube vs NT3-gel, ANOVA with Bonferroni, variance homogeneous)
0.023127 (3 mo, M: LC vs NT3-tube, ANOVA with Bonferroni, variance homogeneous) (14,2,12)
<0.0001 (3 mo, M: LC vs NT3-gel, ANOVA with Bonferroni, variance homogeneous)
0.005832 (3 mo, M: NT3-tube vs NT3-gel, ANOVA with Bonferroni, variance homogeneous)
<0.0001 (3 mo, C2: LC vs NT3-tube, ANOVA with Bonferroni, variance homogeneous) (14,2,12)
<0.0001 (3 mo, C2: LC vs NT3-gel, ANOVA with Bonferroni, variance homogeneous)
1.000000 (3 mo, C2: NT3-tube vs NT3-gel, ANOVA with Bonferroni, variance homogeneous)
0.003409 (3 mo, C5: LC vs NT3-tube, ANOVA with Bonferroni, variance homogeneous) (14,2,12)
0.039472 (3 mo, C5: LC vs NT3-gel, ANOVA with Bonferroni, variance homogeneous)
0.616441 (3 mo, C5: NT3-tube vs NT3-gel, ANOVA with Bonferroni, variance homogeneous)

Fig S7a R
0.295041 (Uninjured, LC vs chronic 6 mo, two-tailed Independent Sample T-Test, variance homogeneous) 8
0.692543 (chronic, LC vs chronic 6 mo, two-tailed Independent Sample T-Test, variance homogeneous) 8
0.105728 (7 d, LC vs chronic 6 mo, two-tailed Independent Sample T-Test, variance homogeneous) 8
0.107258 (1 mo, LC vs chronic 6 mo, two-tailed Independent Sample T-Test, variance homogeneous) 8
0.195607 (3 mo, LC vs chronic 6 mo, two-tailed Independent Sample T-Test, variance homogeneous) 8
C
0.278997 (Uninjured, LC vs chronic 6 mo, two-tailed Independent Sample T-Test, variance homogeneous) 8
0.284225 (chronic, LC vs chronic 6 mo, two-tailed Independent Sample T-Test, variance homogeneous) 8
0.497145 (7 d, LC vs chronic 6 mo, two-tailed Independent Sample T-Test, variance homogeneous) 8

	0.083969 (1 mo, LC vs chronic 6 mo, two-tailed Independent Sample T-Test, variance homogeneous)	8
	0.138966 (3 mo, LC vs chronic 6 mo, two-tailed Independent Sample T-Test, variance inhomogeneous)	5.714683
Fig S7b	0.028531 (Rostral, NT3-tube vs chronic 6 mo, two-tailed Independent Sample T-Test, variance homogeneous)	8
	0.023867 (Caudal, NT3-tube vs chronic 6 mo, two-tailed Independent Sample T-Test, variance homogeneous)	8
	0.037539 (Rostral, NT3-gel vs chronic 6 mo, two-tailed Independent Sample T-Test, variance homogeneous)	8
	0.015509 (Caudal, NT3-gel vs chronic 6 mo, two-tailed Independent Sample T-Test, variance homogeneous)	8
